# Eu^3+^ Site Distribution and Local Distortion of Photoluminescent Ca_3_WO_6_:(Eu^3+^, K^+^) Double Perovskites as High‐Color‐Purity Red Phosphors

**DOI:** 10.1002/advs.202302559

**Published:** 2023-09-26

**Authors:** Takahito Otsuka, Ryohei Oka, Tomokatsu Hayakawa

**Affiliations:** ^1^ Field of Advanced Ceramics Department of Life Science and Applied Chemistry Graduate School of Engineering Nagoya Institute of Technology Gokiso, Showa Nagoya Aichi 466‐8555 Japan; ^2^ Frontier Research Institute for Materials Science (FRIMS) Nagoya Institute of Technology Gokiso, Showa Nagoya Aichi 466‐8555 Japan

**Keywords:** double perovskites, Eu^3+^ red phosphors, fluorescence line‐narrowing, local distortions, rietveld analysis

## Abstract

In this study, Eu^3+^ and K^+^ co‐doped Ca_3_WO_6_ double perovskite, a high‐color‐purity red phosphor, is quantitatively investigated using synchrotron X‐ray diffraction Rietveld refinement, energy dispersive X‐ray spectroscopy, and high‐resolution PL spectroscopy. The Eu^3+^ fluorescence line‐narrowing (FLN) results, used to estimate the Eu^3+^ occupation at given sites (so‐called A and B sites) in the host crystal (A_2_BMO_6_; A, B = Ca; M = W), reveal that the Eu^3+^ ions have a twin distribution in both the A and B sites with high asymmetry ratios of Λ_*A*_ = 9.7 and Λ_*B*_ = 10.7, respectively. More interestingly, at lower Eu^3+^ doping levels, the ions are predominantly located at the B sites (≈75%), indicating that the high color purity of Ca_3_WO_6_:(Eu^3+^, K^+^) is mainly caused by the high asymmetry ratios of the Eu^3+^ sites. Rietveld refinement combined with the FLN technique provides more reliable results for increasing the Eu^3+^ substitution at the A site with Eu^3+^ and K^+^ doping concentrations, which lower the lattice energy of Ca_3_WO_6_:(Eu^3+^, K^+^). The structural distortions owing to K^+^ co‐doping in terms of the quadratic elongation and bond‐angle variance exhibit good correlations with the Ca(Eu)O_12_ A‐site cuboctahedra and Ca(Eu)O_6_ B‐site octahedra, partially accounting for the higher Λ values.

## Graphical abstract

In this study, synchrotron X‐ray diffraction, Rietveld refinement, and high‐resolution photoluminescence spectroscopy are combined to reliably estimate Eu^3+^ positioning and local site distortion in Eu^3+^ and K^+^ co‐doped Ca_3_WO_6_ double perovskites, elucidating their high‐color‐purity red photoluminescence and a twin distribution of Eu^3+^ ions at sites A and B. The computational approach is also adapted to validate the estimations.

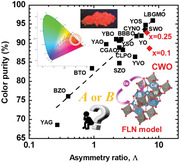

## 1 Introduction

Ultraviolet (UV) or blue‐light‐excited phosphor‐conversion‐type light‐emitting diodes (pc‐LEDs) have attracted increasing attention as third‐generation white‐light illumination devices owing to their high efficiency, high brightness, long lifetime, and environmental protection.^[^ ^1^ ^]^ Commercial white pc‐LEDs generally combine blue LED excitation with yellow phosphors or near‐ultraviolet (NUV) (365–410 nm) LED excitation with red–green–blue (RGB) tricolor phosphors.^[^ ^2^, ^3^, ^4^ ^]^ However, the former type of LEDs are not ideal for indoor illumination because they have a lower color‐rending index (Ra < 80) owing to insufficient red‐luminescent components.^[^ ^5^ ^]^ Conversely, for the latter type of LEDs, the red‐component luminescence intensity of red phosphors excited by NUV LEDs, such as Y_2_O_2_S:Eu^3+^ and Y_2_O_3_:Eu^3+^, is much lower than that of other color components, such as green‐luminescent ZnS:Cu^+^,Al^3+^, and blue BaMgAl_10_O_17_:Eu^2+^ phosphors, which cause the white light to deviate from natural light.^[^ ^6^, ^7^ ^]^ Therefore, new red phosphors that are cost‐effective, have high emission intensities under NUV‐blue excitation, high color purity, and satisfactory chemical stability are required. A relatively new application of red phosphors has been developed in artificial plant factories, where red illumination is used to grow a variety of plants in well‐organized in‐house environments free from diseases and unaffected by unexpected weather conditions.^[^ ^8^, ^9^ ^]^

Recently, Eu^3+^‐adopted molybdate and tungstate compounds have attracted significant attention as potential red phosphors owing to the energy transfer from MO_4_^2−^ (M = W, Mo) tetrahedra or MO_6_^6−^ octahedra to Eu^3+^ ions,^[^ ^10^, ^11^ ^]^ as molybdate and tungstate have a broad, strong charge‐transfer band (CTB) in the NUV‐blue region. Among them, double perovskite oxides A_2_BMO_6_ (A = Ca, Sr, Ba; B = Ca, Sr; M = W, Mo), Eu^3+^, AA'BMO_6_ (A = Na, Li; A’ = Y, La, Gd; B = Mg; M = W, Mo), and Eu^3+^ systems with an MO_6_ octahedral framework have been widely investigated.^[^ ^6^, ^7^, ^10^, ^11^, ^12^, ^13^, ^14^, ^15^

Strong Eu^3+^ red luminescence is caused by the *f*–*f* transition (^5^D_0_–^7^F_J_, J = 0, 1, 2, 3, 4, 5, 6), and the main optical transitions are the magnetic dipole (MD; ^5^D_0_ – ^7^F_1_) transition at ≈594 nm and electric dipole (ED; ^5^D_0_–^7^F_2_) transition at ≈615 nm,^[^ ^16^ ^]^ which are known as the allowed and forbidden transitions, respectively. Based on the Judd–Ofelt theory,^[^ ^17^, ^18^ ^]^ forbidden transitions resulting from the Laporte rule are partially allowed when the activator ions are situated in non‐inversion center sites by the interaction between odd‐ordered terms of crystal fields and electron wave functions,^[^ ^19^, ^20^, ^21^ ^]^ potentially providing a higher ^5^D_0_‐^7^F_2_ transition probability. Therefore, local structural distortion around the Eu^3+^ ions in the host crystal is required to obtain a higher red‐emission intensity and color purity of the phosphor radiation. For this purpose, the ratio of the emission intensity (Λ) for *I* (*^5^D_0_ – ^7^F_2_*) to *I* (*^5^D_0_ – ^7^F_1_*), i.e., the asymmetry ratio, is used to gain a deeper understanding of the local structural distortion around Eu^3+^ because the MD intensity is unaffected by local site symmetry,^[^ ^22^, ^23^, ^24^ ^]^ where *I* (*^5^D_0_ – ^7^F_1_*) and *I* (*^5^D_0_ – ^7^F_2_*) are the emission intensities of the ^5^D_0_ – ^7^F_1_ (MD) and ^5^D_0_ – ^7^F_2_ (ED) transitions, respectively.

As a modern technology, super‐ordering‐structure analysis has recently been developed in materials science fields such as X‐ray holography. This sophisticated technique helps elucidate the local structure around a targeted cation to reveal a unique local structure related to the physical properties.^[^ ^25^, ^26^ ^]^ A spectroscopic approach for investigating the local structures of luminescent centers would also be beneficial. Fluorescence line‐narrowing (FLN) spectroscopy^[^ ^27^, ^28^, ^29^ ^]^ is a well‐known method for obtaining site‐selective photoluminescence (PL) and high‐resolution photoluminescence excitation (PLE) spectra, especially for Eu^3+^ phosphors with multiple Eu^3+^ lattice sites in host materials. This method utilizes a wavelength‐tunable laser as the excitation light source, which provides spectral data that are quite different from those obtained using monochromatized light from a broad, incoherent lamp.^[^ ^30^ ^]^ Owing to the very narrow nature of light from wavelength‐tunable lasers, the excitation of Eu^3+^ at a single site in the host material is possible. Hence, FLN spectroscopy was first applied in the 1970s and has been recognized as a powerful tool for determining site‐selective luminescence properties and Eu^3+^ distribution in multisite dopant phosphors.^[^ ^29^, ^31^, ^32^, ^33^, ^34^ ^]^

The crystal structures and basic optical properties of Eu^3+^‐doped Ca_3_WO_6_ and Ca_3_Mo_x_W_1‐x_O_6_ double‐perovskite phosphors have been studied,^[^ ^10^, ^13^, ^35^ ^]^ owing to their high color purity and emission intensity under NUV or blue‐light excitation. According to these studies, high emission intensity and asymmetry ratios are associated with the substitution of Eu^3+^ ions at the Ca^2+^ sites, referred to as the A and B sites, in the double perovskite structure. The A site has a low site symmetry of *C*_1_, while the B site has a low site symmetry of *C*_i_. Specifically, the substitution of Eu^3+^ and K^+^ for the A site, which exhibits lower symmetry, is suggested to result in luminescence with high color purity, based on Raman spectra and theoretical studies conducted by Zhao and his colleagues.^[^ ^35^ ^]^ However, none of these studies have provided critical data for directly observing the distribution of Eu^3+^ ions between the A and B sites, or for determining if there are differences in the luminescence properties between these sites. In this study, the distribution of Eu^3+^ ions between the A and B sites, as well as the site dependence of luminescence properties, are observed using high‐resolution PLE spectroscopy and site‐selective PL spectroscopy of Eu^3+^ and K^+^‐doped Ca_3_WO_6_ phosphors with varying doping concentration (Sections 2.3–2.5). Furthermore, the lattice energy, electrostatic site potential, and geometrical parameters are estimated by refining the crystal structure using synchrotron X‐ray diffraction (XRD), in order to investigate the structural evolution caused by Eu^3+^ and K^+^ doping (Sections 2.5–3.2). In addition to these in‐depth analyses, the relationship between red color purity and the asymmetry ratio is examined. Basic luminescence properties of PL/PLE spectra, quantum efficiency, and color purity of Eu^3+^ and K^+^‐doped Ca_3_WO_6_ phosphors are also presented (Section 2.2)

## 2 Results

### 2.1 Crystal Structure Determination of Eu^3+^ and K^+^ Co‐Doped Ca_3_WO_6_ Phosphors

**Figure 1:**
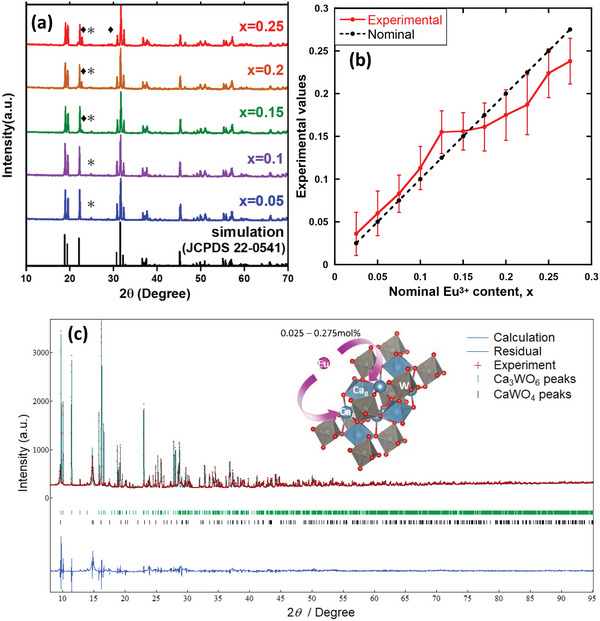
a) XRD patterns of Ca_3‐2x_Eu_x_K_x_WO_6_ (x = 0.05–0.25); b) the number of Eu atoms in each sample, as measured using EDS at three points (error bars include ±2σ); and c) XRD pattern fitted by the Rietveld refinement of Ca_2.95_Eu_0.025_K_0.025_WO_6_ (Eu^3+^, K^+^). The asterisk * denotes CaWO_4_, and ♦ represents Ca_9_Eu_2_W_4_O_24_. The inset shows the crystal structure of monoclinic Ca_3_WO_6_.

The XRD patterns of Ca_3‐2x_Eu_x_K_x_WO_6_ (x = 0.05–0.25) are shown in **Figure** **1**a. These patterns matched well with the monoclinic double‐perovskite structure of Ca_3_WO_6_ (JCPDS 22–0541). In addition, several impurity phases, CaWO_4_ (JCPDS 85–0443) (*) and Ca_9_Eu_2_W_4_O_24_ (JCPDS 05–6230), were confirmed. The Ca_9_Eu_2_W_4_O_24_ phase, including Eu^3+^ ions as the main component, appeared in samples with x > 0.15, whereas the CaWO_4_ phase appeared in all samples. Li et al. established that decreasing the Ca/W ratio induces the formation of the CaWO_4_ phase.^[^ ^36^ ^]^ In this study, the same reasoning can be applied to the emergence of the CaWO_4_ phase.

Figure 1b shows the average number of Eu atoms in each sample based on three points, as measured using EDS; the error bars include±2σ. The actual Eu^3+^ contents in the samples are almost ideal, with slightly lower values observed in x > 0.15. This decrease indicates a deviation from stoichiometry, which can be attributed to the evaporation of K^+^ elements during the high‐temperature sintering process. The detailed crystal structure was determined using Rietveld refinement. Figure 1c shows the XRD pattern fitted via Rietveld refinement using the RIETAN‐FP of Ca_2.95_Eu_0.025_K_0.025_WO_6_. The crystallographic data for Ca_3_WO_6_ (JCPDS 22–0541) were adapted from the initial crystal structure. EDS elemental analysis was used to determine the relative contents of Ca, W, Eu, and K, which provided the site occupations of the main constituent elements (Ca, W, and K) and the total Eu^3+^ ion content in the unit cell (Table S1, Supporting Information). The Eu^3+^ distribution (sites A or B) was determined using FLN spectroscopy (see Section 2.3). An A‐site substitution was adopted for K^+^ ions. Using density functional theory (DFT), Zhao et al.^[^ ^35^ ^]^ calculated the formation energies of Eu^3+^ and K^+^ co‐doped Ca_3_WO_6_ when K^+^ and Eu^3+^ were substituted at the A or B sites of Ca_3_WO_6_. According to the results, both Eu^3+^ and K^+^ with A‐site substitution had the lowest formation energies. Rietveld analysis revealed that oxygen vacancies were also adopted to maintain the charge balance of the unit cell because the K^+^ ions were easily volatilized at higher temperatures during the synthesis process; correspondingly, oxygen could be removed from the perovskite‐type crystal owing to the low oxygen‐site potential.^[^ ^37^ ^]^ Nevertheless, the Rietveld results demonstrated that lattice parameters with lower *R* factors were obtained by considering the Eu^3+^ distribution to the A and B sites and including the experimentally acquired cation composition and oxygen vacancies in the hypothesis. As mentioned above, in the Rietveld refinement, the Ca_3‐2x_Eu_x_K_x_WO_6_ samples were modeled as monoclinic structures with space group *P*2_1_/*c* (No. 14). The lattice parameters for x = 0.025 were determined to be *a* = 5.54597(9) Å, *b* = 5.81112(4) Å, *c* = 9.71981(16) Å, *β* = 124.5810(10) °, and *V* = 257.9087(70) Å^3^ by refinement and finally converged to *R*_wp_ = 5.819%, *R*_e_ = 5.775%, *S* = 1.0077, *R*_B_ = 8.932%, and *R_F_* = 4.253% (**Table** **1**). The analysis was applied to other samples to obtain improved XRD fittings and determine the atomic positions of the Ca_3‐2x_Eu_x_K_x_WO_6_ samples.

**Table 1:** Structure parameters and reliability factors obtained by Rietveld refinement for Ca_2.95_Eu_0.025_K_0.025_WO_6_.

a = 5.54597(9) Å		b =5.81112(4) Å		c = 9.71981(16) Å		
*β* = 124.5810(10) °		*V* = 257.9087(70) Å^3^				
*R*_wp_ = 5.819, *R*_e_ = 5.775, *S* = 1.0077, *R*_B_ = 8.932, *R_F_* = 4.253						
Atom	Site	g	x	Y	z	B(Å^2^)
W	2a	1	0	0	0	0.4
Ca(A)	4e	0.9628	0.26955(98)	0.55667(55)	0.25540(51)	0.8
Ca(B)	2d	0.9628	1/2	0	1/2	0.6
O1	4e	0.9905	0.23079(274)	0.26829(212)	0.06728(175)	1.0
O2	4e	0.9905	0.66488(291)	0.33155(202)	0.44865(175)	1.0
O3	4e	0.9905	0.09143(227)	‐0.04930(227)	0.21662(132)	1.0
Eu(A)	4e	0.0069	0.26955	0.55667	0.25540	0.8
Eu(B)	2d	0.0243	1/2	0	1/2	0.6
K	4e	0.0009	0.26955	0.55667	0.25540	0.8

### 2.2 Photoluminescence Properties

**Figure 2:**
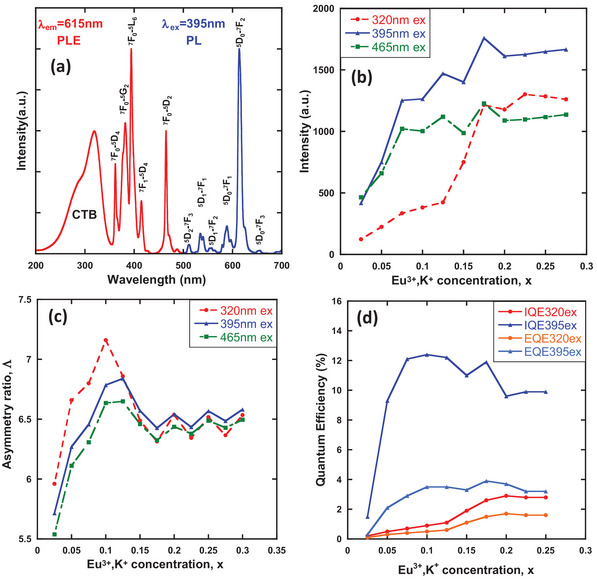
a) PL and PLE spectra of Ca_2.95_Eu_0.025_K_0.025_WO_6_; b) emission intensities of Ca_3‐2x_Eu_x_K_x_WO_6_ (x = 0.025–0.275) at 615 nm under 320, 395, and 465 nm excitation; c) asymmetry ratio, Λ; and d) quantum efficiency of Ca_3‐2x_Eu_x_K_x_WO_6_ as a function of Eu^3+^ and K^+^ concentrations.

**Figure** **2**a shows the PL and PLE spectra of Ca_2.95_Eu_0.025_K_0.025_WO_6_. The shapes of the excitation spectra for the other samples did not change significantly. The sharp excitation peaks of the Eu^3+^ ions (360–500 nm) are assigned to the *f*–*f* transitions of the 4*f* ^6^ configuration, namely, ^7^F_0_–^5^D_4_, ^7^F_0_–^5^G_2_, ^7^F_0_–^5^L_6_, ^7^F_1_–^5^D_4_, and ^7^F_0_–^5^D_2_.^[^ ^38^ ^]^ In addition, a broadband peak at approximately 320 nm corresponds to the charge transfer band (CTB) from the WO_6_ octahedra to Eu^3+^ ions.^[^ ^13^, ^35^, ^39^ ^]^ The wavelength of the CTB is known to be determined by the coordination structure of the W cation within the crystal matrix.^[^ ^40^ ^]^ The appearance of the CTB at a similar position to other Eu^3+^‐doped phosphors with WO_6_ suggests an excitation energy transfer from the WO_6_ octahedron to neighboring Eu^3+^ ions. Specifically, it involves the electronic transition from the 2*p* orbital of O^2−^ in the WO_6_ octahedron to the 4*f* orbital of the Eu^3+^ ions or the *d*‐orbital of the W^6+^ ions, resulting in excited states of Eu^3+^ within the 4f electronic configuration.^[^ ^13^, ^35^, ^41^ ^]^ Ye et al. confirmed that the CT of the WO_6_ octahedra to Eu^3+^ ions was derived from an exchange mechanism, which was first proposed by Dexter in 1953.^[^ ^12^, ^42^ ^]^ In addition, their results supported the energy transfer of WO_6_ octahedra to Eu^3+^ ions in a double‐perovskite‐structure host material, as discussed by Blasse.^[^ ^43^, ^44^ ^]^ The peak at 395 nm (^7^F_0_–^5^L_6_ transition) was the strongest in the absorption spectrum and was available for the excitation of Eu^3+^ ions in the NUV region. This indicates that Ca_3_WO_6_:Eu^3+^ is a good candidate for use as a red phosphor in NUV‐excited white pc‐LEDs.

The sharp emission peaks ranging from 500 to 670 nm were attributed to the well‐known *f*–*f* transitions in the 4f^6^ configuration, namely, the ^5^D_2_–^7^F_3_, ^5^D_1_–^7^F_1_, ^5^D_1_–^7^F_2_, ^5^D_0_–^7^F_1_, ^5^D_0_–^7^F_2_, and ^5^D_0_–^7^F_3_ transitions of Eu^3+^.^[^ ^38^ ^]^ Among them, the most intense peak at 615 nm was assigned to the parity‐forbidden ED ^5^D_0_–^7^F_2_ transition. In contrast, the emission peak at 590 nm, arising from the parity‐allowed MD ^5^D_0_–^7^F_1_ transition, appeared weaker, indicating that the positioning of Eu^3+^ ions in a lattice site lacking an inversion center. Furthermore, the consideration of transition probabilities due to electric and magnetic dipoles is crucial when analyzing the PL intensity. As mentioned in the Introduction, the Laporte rule states that ED transitions (ED: ***d *** =  *e**r***, where *e* represents electronic charge and ***r*** denotes position vector) are not allowed due to their change in sign under inversion operation (〈4*f^N^* |*e**r*** |4*f^N^* 〉 =   − 〈4*f^N^* |*e**r*** |4*f^N^* 〉 under ***r*** → −***r*** in inversion operation), resulting in 〈4*f^N^* |*e**r*** |4*f^N^* 〉 =  0). Conversely, MDs are not affected by inversion operations, which means the MD transition probability between 4*f^N^* electronic states is typically weak but not always zero.^[^ ^44^, ^45^, ^46^ ^]^ The ^5^D_0_‐^7^F_1_ transition of the Eu^3+^ ion represents a purely MD nature and contributes to the orange‐colored PL. According to the Judd‐Ofelt theory,^[^ ^17^, ^18^ ^]^ rare‐earth ions experience crystal fields due to coordination anions, and the odd components of these crystal fields affect the wavefunctions of 4*f^N^* electronic states. They allow for the mixing of these states with opposite parity states, such as *d* and *g* states, which have higher energy levels. As a result, new eigenstates for the ground and excited states of rare‐earth ions are formed within the crystal. Consequently, the transition probability of ED transitions between 4*f^N^* electronic states of rare earth ions in crystals becomes non‐zero. These odd components of the crystal fields are present in the non‐inversion symmetric environment around the Eu^3+^ site. Therefore, the exceptionally high PL intensity observed for the ED nature of the ^5^D_0_‐^7^F_2_ transition can be attributed to the asymmetric site of Eu^3+^ ions, as demonstrated in this study on Ca_3_WO_3_:Eu^3+^,K^+^ phosphors. This explains why the parity‐forbidden ED ^5^D_0_‐^7^F_2_ transition appears more intense in comparison to the parity‐allowed MD ^5^D_0_‐^7^F_1_ transition.

The tungstate ion is well‐known as a luminescence center in inorganic materials. In the case of the Ca_3_WO_6_ compound, the [WO_6_]^6−^ octahedra exhibit a broad PL peak caused by CT centered at 2.91 eV (≈426 nm).^[^ ^39^ ^]^ However, at room temperature (R.T.), the PL intensity originating from [WO_6_]^6−^ is very low because of thermal quenching. Additionally, such a broad PL from [WO_6_]^6−^ was not observed in this study. Therefore, the PL contribution from [WO_6_]^6−^ in our compounds appears to be negligible.

Figure 2b shows the Eu^3+^ and K^+^ concentration dependence of the emission intensities of Ca_3‐2x_Eu_x_K_x_WO_6_ (x = 0.025–0.275) at 615 nm excited at 320 nm (CTB), 395 nm, and 465 nm (direct excitation of Eu^3+^). The evolution of the latter two direct excitations (395 and 465 nm) is almost identical. They displayed a rapidly increasing intensity up to x = 0.075, followed by a moderate increase, and thereafter, an intensity decrease owing to concentration quenching in a concentration region higher than x = 0.175. By contrast, the CTB excitation induced a different evolution that continuously increased up to x = 0.175 and thereafter showed a moderate enhancement. This can be explained by the difference in the local structures of Eu^3+^ ions, which are excitable at their respective excitation wavelengths: when using the CTB excitation wavelength, Eu^3+^ ions at the B site should be excited because the corner sharing via oxygen between WO_6_ and the neighboring EuO_6_ at the B site allows energy transfer from the WO_6_ octahedra to Eu^3+^ ions by an exchange mechanism; consequently, the emission of the B‐site Eu^3+^ ions is more easily induced than that of the A‐site Eu^3+^ ions in double‐perovskite host materials. Moreover, the different evolutions shown in Figure 2b imply that Eu^3+^ ions occupy two different sites (A and B) in the Ca_3_WO_6_ structure.

The asymmetry ratio Λ of Ca_3‐2x_Eu_x_K_x_WO_6_ as a function of Eu^3+^ and K^+^ concentrations is shown in Figure 2c. The asymmetry ratio Λ was calculated using Equation 1.^[^ ^22^ ^]^

(1) $$\;\Lambda =\frac{\int I_{5D0-7F2}}{\int I_{5D0-7F1}}$$

where I_5D0 – 7F1_ and I_5D0–7F2_  are the emission intensities of ^5^D_0_–^7^F_1_ (MD) and ^5^D_0_–^7^F_2_ (ED), respectively. The asymmetry ratio Λ might be useful for discussing the magnitude of the degree of distortion of the Eu^3+^–O^2−^ coordination polyhedron. The tendency of the symmetry ratio of Ca_3‐2x_Eu_x_K_x_WO_6_ as a function of Eu^3+^ and K^+^ concentration differed between the CTB and Eu^3+^ direct excitations, as shown in Figure 2c. This result indicates that the Eu^3+^ ions occupying sites A and B have different asymmetry ratios, particularly at concentrations lower than x = 0.125. In the present Ca_3‐2x_Eu_x_K_x_WO_6_ system, the asymmetry ratio increased with the concentrations of Eu^3+^ and K^+^ up to x = 0.1 and 0.125, respectively, at CTB and direct excitations of Eu^3+^; thereafter, a drop was observed, and the Λ value was maintained. Zhao et al.^[^ ^35^ ^]^ reported an increase in the asymmetry ratio with K^+^ ion doping. They attributed this to the difference in the radii of Ca^2+^ (148 pm) and K^+^ (178 pm) ions. Indeed, the different radii of the host and charge‐compensation ions induces lattice distortion, increasing the asymmetry ratio. Conversely, the decrease in the asymmetry ratio exceeding x = 0.1 and 0.125 indicates that significant doping of the charge‐compensation ion with different ionic radii would relieve the distortion in the lattice (see Section 3.2).

In addition, the asymmetry ratio shown in the system excited by CTB was found to be sufficiently high, i.e., Λ = 7.2 at x = 0.1; this was a higher value in comparison with other red phosphor candidates for pc‐LEDs, such as Y_2_O_3_:Eu^3+^ nanopowder (Λ = 6.3),^[^ ^22^ ^]^ YVO_4_:Eu^3+^ nanopowder (Λ = 8.3),^[^ ^22^ ^]^ BaTiO_3_:Eu^3+^ (Λ = 6.5),^[^ ^47^ ^]^ and BaZrO_3_:Eu^3+^ (Λ = 7.8).^[^ ^48^ ^]^ These values resulted from the distortion induced in the nanoscale particles and/or by the presence of defects such as oxygen vacancies.

The internal quantum efficiency (IQE) and external quantum efficiency (EQE) at Eu^3+^ and K^+^ concentrations of 320 and 395 nm, respectively, were estimated, as shown in Figure 2d. The IQE and EQE, excited at 395 nm, reached 12.3% (x = 0.1) and 3.9% (x = 0.175), respectively. The quantum efficiencies reached their maxima or slightly decreased at higher concentrations, after initially reaching a maximum, and are comparable to those shown in Figure 2b. The high quantum yields at low doping concentrations are attributed to the enhanced asymmetry ratio around x = 0.1, as depicted in Figure 2b. However, at higher concentrations beyond x = 0.175 under 395 nm (direct *f*−*f*) excitation, concentration quenching of PL occurs due to energy killer sites that capture the excitation energy transferred among Eu^3+^ ions.^[^ ^31^, ^49^ ^]^ Nevertheless, the increasing concentration of Eu^3+^ ions compensates for the decrease and thus the PL intensity remains high. Notably, the IQE and EQE excited at 320 nm (CTB) were quite low compared with those excited at 395 nm at maxima of 2.9% (x = 0.2) and 1.9% (x = 0.2), respectively. In the quantum‐efficiency experiment, the absorption ratio at the corresponding wavelength, defined as the number of absorption photons divided by the number of incident photons, was estimated, as shown in Figure S1 (Supporting Information). The present series of samples had almost twice the absorption ratio at 320 nm (CTB) excitation than at 395 nm (*f*−*f* direct) excitation. Nevertheless, the quantum efficiencies (IQE and EQE) for the 320 nm excitation were much lower than those for direct excitation. It should be noted that Eu^3+^ PL incited under 320 nm excitation required energy transfer from O_2p_ electrons to Eu^3+^ ions, indicating the possibility of an energy trap for defects during the energy‐transfer process and/or non‐radiative relaxation of O_2p_ electrons. Moreover, energy loss may occur within Eu^3+^ ions after receiving excitation energy from O_2p_ electrons; consequently, Eu^3+^ ions would relax to the ground state nonradiatively. Host absorption is also possible because the reported bandgap of the Ca_3_WO_6_ crystal is 3.82 eV,^[^ ^50^ ^]^ corresponding to the absorption edge wavelength of ≈325 nm; therefore, this is one of the loss processes of the absorbed photons.

Using the Blasse equation,^[^ ^51^ ^]^ the critical distance for PL concentration quenching was estimated as follows:

(2) $$R_{c}=2{\left[\frac{3V}{4\pi x_{c}Z}\right]}^{1/3}$$

where *V* is the unit cell volume, *x_c_* is the critical concentration for PL quenching, and *Z* is the number of cation sites in the unit cell. At *x* = 0.225 and 0.175, the PL intensity was maximized in the CTB and direct excitation, respectively, and thereafter decreased with a further increase in the concentration. The *R_c_* values were estimated as 11.0 and 11.5 Å. Blasse^[^ ^51^ ^]^ suggested multipole and exchange interactions as the mechanisms of energy transfer for *R*_c_ > 5 Å and *R*_c_ < 5 Å, respectively. The *R*_c_ values exceed 5 Å, indicating that the mechanism between Eu^3+^ ions in the CaWO_3_ double perovskite was likely caused by multipole interactions.

**Figure 3:**
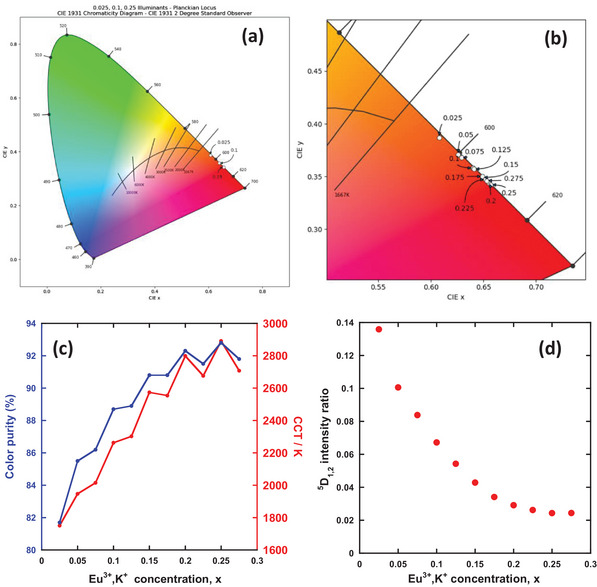
CIE chromaticities of Ca_3‐2x_Eu_x_K_x_WO_6_ phosphors. a) Overall picture and b) zoomed‐in view. Concentration dependence of c) the color purity of red PL and d) blue‐green PL component assignable to ^5^D_1,2_ emissions for Ca_3‐2x_Eu_x_K_x_WO_6_ phosphors.

The Commission Internationale de l’Éclarirage (CIE) chromaticity coordinates of Ca_3‐2x_Eu_x_K_x_WO_6_ are shown in **Figure** **3**a,b and listed in **Table** **2**. The correlated color temperatures (CCTs) were also estimated. Based on the CIE results, an increase in Eu^3+^ and K^+^ concentrations results in the color coordinates approaching the ideal red chromaticity (0.67, 0.33) for the National Television Standard Committee (NTSC) system. The CCT values ranged from 2000 to 3000 K, except at x = 0.025 and 0.05. To assess the effect of Eu^3+^ and K^+^ co‐doping, the color purity (*C.P*.) was calculated as follows:

(3) $$C.P.=\frac{\sqrt{{\left(x_{s}-x_{i}\right)}^{2}+{\left(y_{s}-y_{i}\right)}^{2}}}{\sqrt{{\left(x_{d}-x_{i}\right)}^{2}+{\left(y_{d}-y_{i}\right)}^{2}}}\times 100\%$$

where (*x_d_*, *y_d_*) are the coordinates of the dominant wavelength, (*x_s_*, *y_s_*) are the coordinates of the sample point, and (*x_i_*, *y_i_*) are the coordinates of the illuminant point. In this study, (*x_d_*, *y_d_*) = (0.680,  0.320)  and (*x_i_*, *y_i_*) = (0.3101,  0.3162)  for a dominant wavelength of 615 nm. The calculation results are listed in Table 2, and Figure 3c reveals that the increasing dopant concentration improved the color purity from 81.7% (x = 0.025) to 92.8% (x = 0.25), which was high in comparison with, for instance, the reported color purity (91.2%) of perovskite‐type Ca_1‐2x_Eu_x_Li_x_TiO_3_ red phosphors.^[^ ^52^ ^]^ As observed in the PL spectra (Figure 2a), the Eu^3+^ in the Ca_3_WO_6_:(Eu^3+^, K^+^) phosphors exhibited ^5^D_2_ and ^5^D_1_ emissions at lower Eu^3+^ concentrations, which reduced the purity of the red color (Figure 3c). Figure 3d shows the spectral components in the blue and green regions, which are assignable to ^5^D_2_−^7^F_3_, ^5^D_1_−^7^F_1_, and ^5^D_1_−^7^F_2_ transitions in comparison with all the spectral components, including red PL. The contribution of the blue‐green PL components was estimated at ≈14% at x = 0.025 and decreased to ≈2% at higher concentrations owing to the cross‐relaxation processes between neighboring Eu^3+^ ions.^[^ ^53^, ^54^ ^]^ This is the reason for the color purity enhancement from 82% to 93% with increasing Eu^3+^ concentration, as shown in Figure 3c.

**Table 2:** CIE coordinates of the red PLs for Ca_3‐2x_Eu_x_K_x_WO_6_ phosphors.

X	CIE x	CIE y	CCT / K	*C.P*. (%)
0.025	0.608	0.387	1750.9	81.7
0.050	0.626	0.371	1946.8	85.5
0.075	0.629	0.368	2015.3	86.2
0.100	0.640	0.358	2262.6	88.7
0.125	0.641	0.357	2302.4	88.9
0.150	0.649	0.349	2573.9	90.8
0.175	0.649	0.350	2554.0	90.8
0.200	0.655	0.344	2798.6	92.3
0.225	0.652	0.347	2676.0	91.5
0.250	0.657	0.342	2891.4	92.8
0.275	0.653	0.346	2707.7	91.8

**Figure 4:**
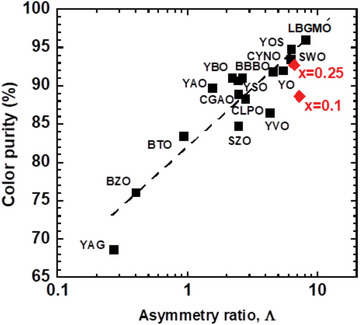
Red‐color purity of Eu^3+^ luminescence as a function of asymmetry ratio. The data for Ca_3‐2x_Eu_x_K_x_WO_6_ are plotted as red diamonds (♦). YAG(Y_2.97_Al_5_O_12_:0.03Eu^3+^),^[^ ^50^ ^]^ (YBO)Y_0.8_BO_3_:0.2Eu^3+^,^[^ ^51^ ^]^ YAO(Y_0.95_AlO_3_:0.05Eu^3+^),^[^ ^52^ ^]^ YVO(Y_0.95_VO_4_:0.05Eu^3+^),^[^ ^53^ ^]^ YO(Y_1.998_O_3_:0.002Eu^3+^),^[^ ^54^ ^]^ YOS(Y_1.88_O_2_S:0.12Eu^3+^),^[^ ^55^ ^]^ BTO(Ba_0.94_TiO_3_:0.06Eu^3+^),^[^ ^56^ ^]^ BZO(BaZr_0.97_O_3_:0.03Eu^3+^),^[^ ^57^ ^]^ SZO(Sr_0.97_ZrO_3_:0.03Eu^3+^),^[^ ^57^ ^]^ CYNO(Ca_2_Y_0.6_NbO_6_:0.4Eu^3+^),^[^ ^58^ ^]^ SWO(Sr_2.4_WO_6_:0.3(K^+^,Eu^3+^)),^[^ ^15^ ^]^ LBGMO(Li_3_Ba_2_Gd_0.6_Eu_2.4_(MoO_4_)_8_),^[^ ^59^ ^]^ BBBO(Ba_3_(Bi_0.5_Eu_0.5_)_2_(BO_3_)_4_),^[^ ^60^ ^]^ YSO(Y_1.95_SiO_5_:0.05Eu^3+^),^[^ ^61^ ^]^ CGAO(CaGd_0.93_AlO_4_:0.07Eu^3+^),^[^ ^62^ ^]^ and CLPO(Ca_9.9_Li(PO_4_)_7_:0.1Eu^3+^).^[^ ^63^ ^]^ The corresponding CIE coordinates are given in Table S2 (Supporting Information).

Here, we attempt to assess the CIE coordinates and *C.P*. values of various Eu^3±^doped phosphors^,[^ ^15^, ^55^, ^56^, ^57^, ^58^, ^59^, ^60^, ^61^, ^62^, ^63^, ^64^, ^65^, ^66^, ^67^, ^68^ ^]^ as a function of the asymmetry ratio, as shown in Table S2 (Supporting Information) and **Figure** **4**, including Ca_3_WO_6_:(Eu^3+^, K^+^), which will provide more insight into the correlation of the *C.P*. value with the asymmetry ratio. As indicated by the linear correlation coefficient (0.750), the correlation is not linear but semi‐logarithmic (correlation coefficient: 0.917) according to *C*.*P*.  =  82.04  +  15.59  ×  log_10_(Λ) for 0.2 < Λ < 10. Nevertheless, this plot clarifies the reason for the higher color purity of the Eu^3+^ red luminescence with an increase in local asymmetry around the Eu^3+^ ions. It can be seen that the *C.P*. values do not always fall on the regression line, which is due to their spectral shapes, PL peak shift, and line broadenings of the ^5^D_0_‐^7^F_J_ signals (J = 0,1, 2, 3, 6) in the red‐orange region. Other previously reported values, for instance, *C.P*. = 82.9%, Λ = 4.61 for YVO_4_:2%Eu^3+^,^[^ ^69^ ^]^ and *C.P*. = 94.2%, Λ = 1.04 for Y_2_O_2_S:0.5%Eu^3+^,^[^ ^70^ ^]^ deviate significantly from the regression line (not plotted in the figure) owing to the influence of blue‐green luminescence from ^5^D_1,2_, as discussed above. Alternatively, these reported values might have been calculated in the limited spectral region of longer wavelengths (orange‐red).

Moreover, the PL color properties observed in this study could be derived from structural modifications or distortions caused by the co‐doping of large monovalent K^+^ ions acting as charge compensators. To further investigate the structural factors influencing the PL properties, high‐resolution PL spectroscopy was performed, and the obtained data were used for the crystallographic analysis of the Rietveld refinement to clarify the effect of the local structures on the macroscopic PL properties. The next section describes the methodology and provides insights into the development of phosphors with high color purity.

### 2.3 High‐Resolution PLE Spectra

**Figure 5:**
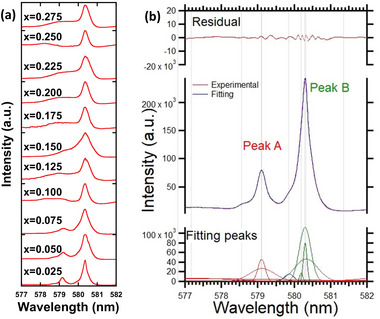
a) High‐resolution PLE spectra of Ca_3‐2x_Eu_x_K_x_WO_6_ (x = 0.025‐0.275) for the red PL peak at 615 nm, measured using the FLN technique; and b) deconvolution of high‐resolution PLE spectrum of Ca_2.95_Eu_0.025_K_0.025_WO_6_ observed at 615 nm.

The PLE spectra of Ca_3‐2x_Eu_x_K_x_WO_6_ (x = 0.025–0.275) and the deconvoluted PLE spectrum of Ca_2.95_Eu_0.025_K_0.025_WO_6_ obtained via the FLN technique at 615 nm are shown in **Figure** **5**a,b, respectively. These spectra are within the ^7^F_0_–^5^D_0_ transition range of 577 (≈17 330 cm^−1^) and 581.4 nm (≈17 200 cm^−1^).^[^ ^31^ ^]^ In the case of Eu^3+^ in a lattice site, the ^7^F_0_–^5^D_0_ transition should give rise to a peak because ^7^F_0_ and ^5^D_0_ are nondegenerate levels. As shown in the figure, two different peaks appear at λ_A_ = 579.1 nm (peak A) and λ_B_ = 580.3 nm (peak B). The multiple peaks appearing in the ^7^F_0_–^5^D_0_ transition indicate the presence of a corresponding number of Eu^3+^ lattice sites. Moreover, the peak positions indicated that the different excitation energies were affected by the intensity of each site in the crystal field. Specifically, it was established that the red shift of the Eu^3+^ *f*–*f* transition occurs in crystals, as opposed to the Eu^3+^ species existing as free ions. Jørgensen^[^ ^71^ ^]^ explained this as the “nephelauxetic effect,” indicating an energy shift of the excitation level by crystal fields. Therefore, the Eu^3+^ ions occupy two different lattice sites, as evidenced by the spectra of the two ^7^F_0_–^5^D_0_ transition peaks. The two Eu^3+^ lattice sites must be Ca^2+^ sites in the Ca_3_WO_6_ structure, where the Ca^2+^ ions occupy the A (12‐coordinate) and B (6‐coordinate) sites in the perovskite structure. Each excitation peak was identified by comparing the A‐ and B‐site crystal‐field potentials, as the excitation peak shift is dependent on the depth of the crystal‐field potential via the nephelauxetic effect. The electrostatic potentials at the A and B sites were estimated as −1.44 and −1.77 *e*Å^−1^, respectively, considering only the nearest neighboring anions in the A‐ and B‐site polyhedra. As a result, the peaks at λ_A_ and λ_B_ were assigned to A‐ and B‐site Eu^3+^ ions, respectively. Deconvolution was applied to the other samples, and the results are shown in Supporting Information. For lower x concentrations, the two main peaks were deconvoluted to determine the integrated intensities of peaks A and B, with a slight background band (Figure S2a–c, Supporting Information). The broad background increased for x  ≥  0.100 (Figure S2d–f, Supporting Information), presumably indicating the presence of defective sites for Eu^3+^ ions (interstitial or surface defects). Moreover, when x  ≥  0.175, additional broad bands separated by spectral deconvolution, as shown in Figure S2g–i (Supporting Information), appeared at ≈580.4 (≈17 230 cm^−1^) and ≈577.7 nm (≈17 310 cm^−1^). These bands seem to be attributed to Eu^3+^ in the impurity crystal phase, Ca_9_Eu_2_W_4_O_24_, as the peak position corresponded to the excitation bands spanning from ≈578.7 to ≈581.4 nm in the high‐resolution PLE spectrum of Ca_9_Eu_2_W_4_O_24_ previously reported by Qin et al.^[^ ^72^ ^]^

The width of peak A increased with increasing Eu^3+^ and K^+^ concentrations. This implies a diversification of the electrostatic potential in the A‐site Eu^3+^ owing to two possible factors: the presence of oxygen vacancies and varied atomic positions in the unit cell. As discussed previously, oxygen vacancies are common in perovskite structures. In addition, according to Shannon et al.,^[^ ^73^ ^]^ the radii of 12‐coordinate Ca^2+^ and K^+^ ions are 148 and 178 pm, respectively, corresponding to a size difference of 30 pm. Thus, the atomic positions of the constituents around the K^+^ ions were affected by K^+^ doping. Based on these two factors, the B‐site polyhedron was tilted while maintaining its shape via strong covalent bonding with oxygen, whereas distortion of the A‐site polyhedron was induced owing to its ionic bonding nature. Thus, we believe that the diversification, particularly at the A site, is caused by slightly different local environments around Eu^3+^ ions, that is inhomogeneous broadening of Eu^3+^ PL. This is attributed to the introduction of oxygen vacancies and the alteration of oxygen positions through Ca^2+^ substitution with K^+^ ions of different ionic radii (refer to Section 3.2).

### 2.4 Site‐Selective PL Spectra

**Figure 6:**
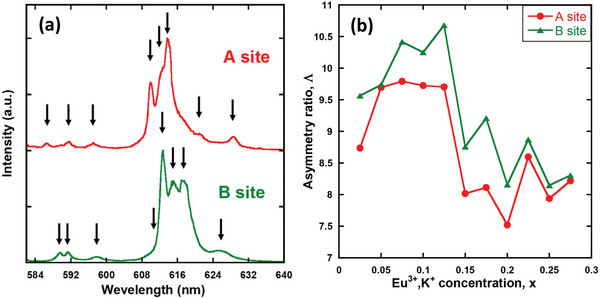
a) A‐ and B‐site‐selective PL spectra of the x = 0.025 sample, and b) site‐selective asymmetry ratio Λ of Ca_3‐2x_Eu_x_K_x_WO_6_ (x = 0.025–0.275) as a function of Eu^3+^ and K^+^ concentrations.

**Figure** **6**a displays the A‐ and B‐site‐selective PL spectra of the sample with x = 0.025. Energy‐sublevel splitting via the crystal‐field effect was observed in the spectra. The splitting values of the ^5^D_0_–^7^F_1_ and ^5^D_0_–^7^F_2_ transition peaks were dependent on the site symmetry.^[^ ^74^ ^]^ The crystallographic data of Ca_3_WO_6_ (JCPDS 22–0541) indicated that the A‐ and B‐site symmetries have a lower site symmetry (*C_1_*) and an inversion center (*C_i_*), respectively. Thus, the ^5^D_0_–^7^F_1_ and ^5^D_0_–^7^F_2_ transition peaks split into a triplet and pentet, respectively, as observed in each spectrum (marked ↓), corresponding to the degeneracies of the final levels of ^7^F_1,2_. Low site symmetries were found to disrupt the degeneracies via the crystal‐field effect.^[^ ^75^ ^]^ The difference between the A and B sites for the Eu^3+^ ions was reflected in the shape of the site‐selective spectra.

Figure 6b shows the site‐selective asymmetry ratio Λ of Ca_3‐2x_Eu_x_K_x_WO_6_ (x = 0.025–0.275) as a function of the Eu^3+^ and K^+^ concentrations. The B‐site asymmetry ratio was higher than that of site A for all samples. The highest Λ value was 10.7 and 9.7 for sites B and A, respectively. The asymmetry ratio tended to increase with x, particularly at site B. When the concentration increased to x = 0.125, the site‐selective asymmetry ratio decreased for both A and B sites. This agrees with the asymmetry ratio evolution estimated from the conventionally obtained PL spectra (Figure 2a). This result indicates that all the Eu^3+^ ions in Ca_3_WO_6_ exhibit high asymmetry ratios, which explains the high color purity of Eu^3+^‐doped Ca_3_WO_6_. The microscopic Λ values were greater than the macroscopic Λ values observed under the excitation of a monochromatic light source, as shown in Figure 2c. As implied by the FLN data, one possible reason for this is the presence of defective sites for Eu^3+^ ions other than sites A and B, which would result in a less symmetric site.

### 2.5 Eu^3+^ Distribution in Ca_3_WO_6_

**Figure 7:**
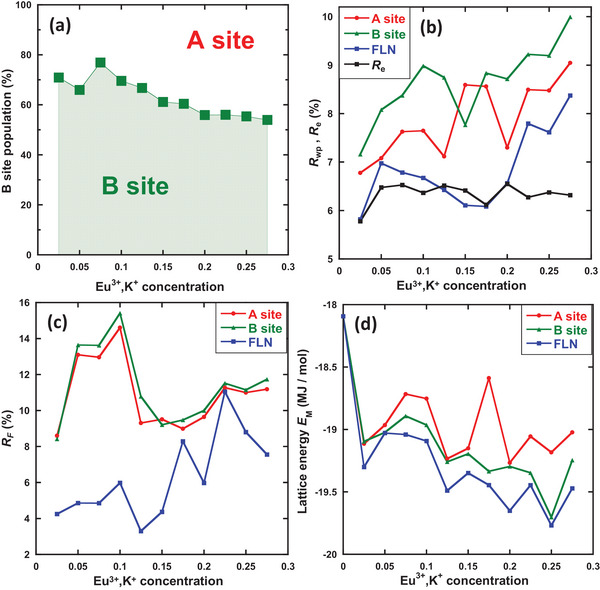
a) Eu^3+^ distribution of A and B sites in Ca_3‐2x_Eu_x_K_x_WO_6_ (x = 0.025–0.275), determined using FLN spectroscopy as a function of Eu^3+^ and K^+^ concentrations. b) *R*_wp_, *R*_e_, and c) *R*_F_ obtained via Rietveld refinement for 100% Eu^3+^ at the A site, 100% Eu^3+^ substitution at the B site, and Eu^3+^ substitution at both sites, with the distribution determined by FLN. d) Lattice energy EM calculated for the crystal structure of Ca_3‐2x_Eu_x_K_x_WO_6_ (x = 0.025–0.275) using VESTA and Rietveld refinement, shown as a function of Eu^3+^and K^+^ concentration.

**Figure** **7**a shows the Eu^3+^ distributions of the A and B sites in Ca_3‐2x_Eu_x_K_x_WO_6_ (x = 0.025–0.275) as a function of the Eu^3+^ and K^+^ concentrations. The following Equations (4), (5), (6), (7), (8) were used to estimate the Eu^3+^ distribution at sites A and B based on the high‐resolution PLE and site‐selective PL spectra.

(4) $$N_{Eu}=N_{A\;site}+N_{B\;site}$$

(5) $$P_{A}=\frac{N_{A\;site}}{N_{Eu}}=\frac{\frac{S_{peak\;A}}{C_{A}}}{\frac{S_{peak\;A}}{C_{A}}+\frac{S_{peak\;B}}{C_{B}}}$$

(6) $$P_{B}=\frac{N_{B\;site}}{N_{Eu}}=\frac{\frac{S_{peak\;B}}{C_{B}}}{\frac{S_{peak\;A}}{C_{A}}+\frac{S_{peak\;B}}{C_{B}}}$$

(7) $$C_{A}=\frac{\Lambda_{A}}{1+\Lambda_{A}}$$

(8) $$C_{B}=\frac{\Lambda_{B}}{1+\Lambda_{B}}$$

where *N*_Eu_ denotes the total number of Eu^3+^ ions; *N*_A site_ and *N*_B site_ denote the number of Eu^3+^ ions at the A and B sites in the Ca_3_WO_6_ structure, respectively; *P*_A_ and *P*_B_ denote the Eu^3+^ ratios at the A and B sites, respectively; and *C*_A_ and *C*_B_ denote the calibration factors for the A‐ and B‐site PL peak areas and *S*_peak A_ and *S*_peak B_ in the PLE spectra, respectively. Notably, the PLE spectra were recorded with a larger slit width (Δλ_em_) of ≈15 nm to collect more ^5^D_0_−^7^F_2_ emissions. As the integrated intensity of the ^7^F_0_–^5^D_0_ excitation band for ^5^D_0_−^7^F_2_ emission depends on the transition probability *W*_0 − 2_ and the number of Eu^3+^ ions at each site, the number of Eu^3+^ ions in the A and B sites should be estimated by eliminating the influence of the transition probabilities *W*_0 − 2_ of each Eu^3+^ ion from the integrated intensity. The calibration factors in Equations 7 and 8 ^[^ ^76^ ^]^ are the branching ratios of the ^5^D_0_−^7^F_2_ emission, *W*_0 − 2_/(*W*_0 − 1_ + *W*_0 − 2_), under the approximation of negligible ^5^D_0_−^7^F_0_ and ^5^D_0_−^7^F_3‐4_ emissions. Thus, the quotient of the integrated ^5^D_0_–^7^F_2_ intensity (*S*_*peak* *A*_ or *S*_*peak* *B*_) divided by the branching ratio was proportional to the number of Eu^3+^ ions at a specific site (see Supporting Information for a detailed derivation). Based on the aforementioned considerations, the Eu^3+^ distribution was analyzed, and the results in Figure 7a reveal that the distribution of Eu^3+^ was ≈77–54% for site B in all samples. The highest B‐site occupation of Eu^3+^ is 77.0% at x = 0.075, decreasing to 54.0% at x = 0.275.

The Eu^3+^ distribution estimated using this method was verified by comparing the reliability factors *R*_wp_ and *R_F_* obtained via Rietveld refinement for Eu^3+^‐doped ions substituted only at site A, only at site B, and at both sites, with a distribution estimated by the FLN spectra, as shown in Figure 7b,c (See also Table S3 in Supporting Information). *R*_wp_ and *R_F_* are reliability factors that indicate the degree of agreement between the fitting and calculation validity of a structural model.^[^ ^77^ ^]^ The *R*_wp_ and *R_F_* results show that the FLN distribution model is more reasonable than the A‐ or B‐site substitution models.

To explain the observed PL properties as a function of Eu^3+^ concentration (Figure 2b), additional discussion is required regarding the dominant Eu^3+^ occupation at the B site at a lower Eu^3+^ concentration and its partial migration to the A site. As mentioned above, the 320 nm excitation was considered to excite the majority of Eu^3+^ ions at the B site owing to the energy‐transfer mechanism of the CTB of O_2p_ in the WO_6_ octahedra, whereas direct *f*−*f* excitation excited Eu^3+^ ions at both the A and B sites. Nevertheless, under 320 nm excitation, the PL intensity was lower than that under direct excitation. However, this observation has already been explained by the possible loss processes occurring during energy transfer from the O_2p_ electron and/or populating the ^5^D_0_ level from higher levels (see the IQE/EQE discussion in the Section 2.2). One may question why concentrations exceeding x = 0.125 induced a more rapid increase in the red PL in the 320 nm excitation (B‐site excitation) compared with those in the direct excitations, even for a higher A‐site distribution of Eu^3+^ ions at higher concentrations. This can possibly be explained by the transfer of energy from the B‐site Eu^3+^ to the A‐site Eu^3+^ after Eu^3+^ excitation at the B site and the subsequent emission of PL under CTB excitation by Eu^3+^ at the A site.^[^ ^78^ ^]^ Fortunately, the A‐ and B‐site Eu^3+^ ions retained higher Λ values (Figure 6b); therefore, the red PL color purity did not deteriorate, even after the energy transfer from Eu^3+^(B) to Eu^3+^(A). This is evidenced by the presence of Eu^3+^–Eu^3+^ interactions that improve color purity by reducing ^5^D_1,2_ emissions via cross‐relaxation between neighboring Eu^3+^ ions with increasing Eu^3+^ concentration, as explained in Section 2.2.

In this study, the following questions arise: Why are the Eu^3+^ ions distributed among the A and B sites, and why is a selected lattice site (A or B) not used for Eu^3+^ ions in the Ca_3_WO_6_ crystal? According to Van Gool and Piken,^[^ ^79^ ^]^ the chemical composition and crystal structure are determined by the thermodynamic energy reflected by Madelung's electrostatic potential. Hoppe^[^ ^80^ ^]^ demonstrated that the enthalpy of formation of solids was reflected by the Madelung electrostatic potential, and Glasser^[^ ^81^ ^]^ showed that the total lattice energy and Madelung electrostatic potential had a linear relationship in ionic solids. Based on these fundamentals, the Madelung lattice energies (*E*_M_) were calculated for the Ca_3‐2x_Eu_x_K_x_WO_6_ (x = 0.025–0.275) crystal structure models obtained via Rietveld refinement of Figure 7b,c. The results shown in Figure 7d can be compared with their structural stabilities. The electrostatic potential (ϕ_*i*_) and *E*_M_ of the crystal structure were calculated using the following equations in the VESTA program,^[^ ^37^, ^82^ ^]^

(9) $$\phi_{i}=\sum_{j}\frac{Z_{j}}{4\pi \varepsilon_{0}l_{ij}}$$

where *Z*_j_ denotes the valence of ion j, ε_0_ is the permittivity of a vacuum, and *l*_ij_ is the distance between ions i and j.

(10) $$E_{M}=\frac{1}{2}\;\sum_{i}\phi_{i}Z_{i}W_{i}$$

where $W_{i}=\frac{\left(\mathrm{occupancy})\times (\mathrm{number}\;\mathrm{of}\;\mathrm{equivalent}\;\mathrm{position}\right)}{\left(\mathrm{number}\;\mathrm{of}\;\mathrm{general}\;\mathrm{equivalent}\;\mathrm{position}\right)}$.

In all cases, the co‐doping of Eu^3+^ and K^+^ at the Ca site (A and/or B) in the Ca_3_WO_6_ lattice stabilized the lattice energy; however, increasing the doping levels was found to induce different behaviors for the A‐site only, B‐site only, and Eu^3+^ A/B distributions obtained from the FLN analysis. The FLN distribution model clearly exhibited the most stable energy compared with the other models. When Eu^3+^ ions were substituted at both A and B sites with the distribution shown in Figure 7a, rather than only at site A or B, the *E*_M_ was stabilized by Eu^3+^ and K^+^ doping. The reason for this stabilization is explained in the next section.

## 3 Discussion

### 3.1 Relationship Between Madelung Electrostatic Potentials and Structural Parameters

**Figure 8:**
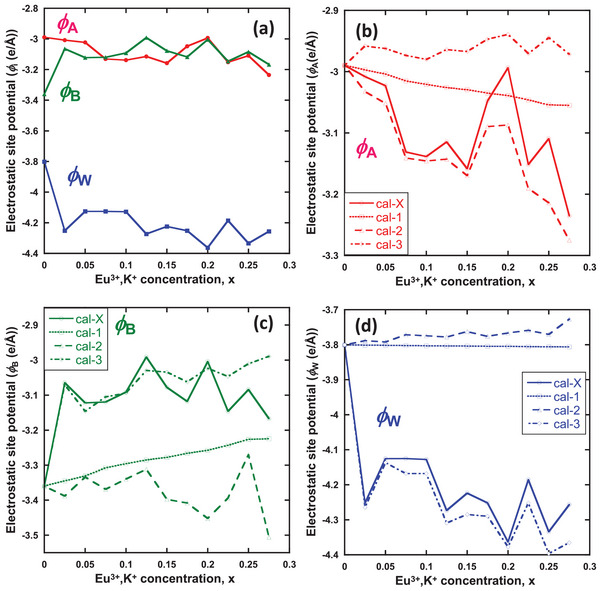
a) Electrostatic site potentials ϕ_*i*_ of A, B, Eu, and W sites, calculated in cal‐X (Table 3). Electrostatic site potentials of b) A‐site Eu^3+^, c) B‐site Eu^3+^, and d) W‐site Eu^3+^ calculated in cal‐1–3, compared with the results of cal‐X.

Yoshimura and Sardar showed that the structural stability of a crystal can be described by Madelung electrostatic site potentials and lattice energies, which are applicable to perovskite‐structured ABO_3_ compounds.^[^ ^37^ ^]^ As defined in Equation 10, the Madelung lattice energy (*E*_M_) is influenced by the site potential, ϕ_*i*_. Therefore, the reason for the stabilization of *E*_M_ by Eu^3+^ and K^+^ doping can be elucidated by comparing the site‐potential evolutions at each lattice site as a function of the Eu^3+^ and K^+^ concentrations. The site potentials shown in **Figure** **8**a were obtained from the parameters of Eu^3+^ occupation determined using FLN spectroscopy (Figure 7a), the chemical composition determined using EDS analysis (Table S1, Supporting Information), and more reliable atomic positions estimated via Reitveld refinement (Table 1), as discussed in the preceding section. ϕ_A_ for the Eu^3+^‐substituted A site and ϕ_W_ for the W site were stabilized by Eu^3+^ and K^+^ doping. By contrast, ϕ_B_ for the Eu^3+^‐substituted B site was destabilized by Eu^3+^ and K^+^ doping. Fundamentally, ϕ_*i*_ is described as a function of two variables: the valence of the ions (*Z_j_*) and distance (*l_ij_*) between Eu^3+^(W^6+^) and neighboring *j*‐th ions, as shown in Equation 9. Therefore, site potentials can be affected by the three scenarios described below.

- Changes in the charge distribution of the structure induced by the substitution of Ca^2+^ with Eu^3+^ and K^+^ affect the lattice site valence.
- Changes in the number of cation or anion vacancies affect the lattice site valence.
- Changes in the atomic positions of all ions by substituting Ca^2+^ with Eu^3+^ and K^+^ affect the distance between the ions.

To formulate scenarios I–III, a series of Eu^3+^ occupation parameters at sites A and B, chemical compositions, and atomic positions in the unit cell were considered and are listed in **Table** **3**. The first set of parameters (cal‐1) was used to observe the effect of the monovalent charge compensator, Ca^2+^(A, B) + Ca^2+^(A) → Eu^3+^(A, B) + K^+^(A). To avoid the vacancy effect, atomic positions were obtained from the database (JCPDS 22–0541). The second set of parameters, denoted cal‐2, reflects the evolution of scenarios I and II. Accordingly, scenario II could be extracted by subtracting ϕ_*i*_, obtained from the cal‐1 parameter set, from the ϕ_*i*_ of cal‐2, where the structure of Ca_3_WO_6_ from the database was used to simplify the calculation. The final set of parameters (cal‐3) reflects scenario III, whose atomic positions were taken from those obtained via Rietveld refinement as a function of the Eu^3+^ and K^+^ concentrations.

**Table 3:** Parameters adopted for Rietveld refinement in cal‐X and cal‐1–3.

	Eu^3+^ occupation	Chemical composition	Atomic positions	Data & Scenarios
cal‐X	from FLN	from EDS	from Rietveld analysis	Figure 8a
cal‐1	from FLN	Ca_3‐2x_K(A)_x_Eu(A,B)_x_WO_6_	from database^a)^	I
cal‐2	from FLN	from EDS	from database^a)^	I and II
cal‐3	Non‐doped	Ca_3_WO_6_	same as cal‐X	III

^a)^ JCPDS 22‐0541.

The calculated site potentials for the A‐site Ca(Eu,K) (ϕ_A_), B‐site Ca(Eu) (ϕ_B_), and W (ϕ_W_) are shown in Figure 8b–d, respectively. Focusing on ϕ_A_ (Figure 8b), cal‐1,2 showed an overall stabilization trend with increasing Eu^3+^ and K^+^ concentrations, similar to the evolution obtained from the experimental parameters (cal‐X). The evolution of cal‐1 appeared to be linear, whereas that of cal‐X and cal‐2 was more complex. By contrast, cal‐3 exhibited a slight destabilization trend. Notably, the cal‐X and cal‐2 results resembled the evolution of ϕ_A_ with an increase in Eu^3+^ and K^+^ concentrations, i.e., ϕ_A_ evolution was strongly dependent on the change in charge distribution, as observed in scenarios I and II. To determine which scenario was more likely to occur for ϕ_A_, we compared the correlation coefficient of the experimental result (cal‐X) with those of scenario I (cal‐1) and scenario II (cal‐2), which was assessed by subtracting the evolution in cal‐1 from that in cal‐2. The correlation coefficients were estimated to be 0.78 (cal‐X vs scenario I) and 0.99 (cal‐X vs scenario II). In conclusion, the ϕ_A_ stabilization must be caused by the introduction of vacancies.

Moreover, the correlation coefficients reflecting the amount of oxygen (or cation) vacancies (mol%) and site potential ϕ_A_ for Eu^3+^ estimated in scenario II (ϕ_A_(cal‐2) – ϕ_*A*_(cal‐1)) were calculated to determine which defective vacancies of oxygen or cations influenced ϕ_A_ stabilization. Robust negative correlations of −0.96 and −0.94 were estimated for cal‐2 and cal‐1, respectively. In addition, the change in charge distribution owing to Eu^3+^ substitution at the A or B sites might affect ϕ_A_. The evolution of ϕ_A_ for the case of complete Eu^3+^ substitution at the A or B sites, as well as K^+^ substitution at the A site (while maintaining the structural parameters (Table S4, Supporting Information)) is shown in Figure S3 (Supporting Information). When the Eu^3+^ ion occupies the A site, ϕ_A_ is remains unchanged because the average valence of the A‐site ions does not vary with doping (2Ca^2+^(A) → Eu^3+^(A) + K^+^(A)). Moreover, ϕ_A_ was stabilized when Eu^3+^ ions were situated at the B site. Significantly, an increase in the B‐site valence together with a decrease in the A‐site valence (Ca^2+^(B) + Ca^2+^(A) → Eu^3+^(B) + K^+^(A)) can reduce the site potential for Eu^3+^. According to Yoshimura and Sardar,^[^ ^37^ ^]^ the site A potential destabilizes with decreasing valence of the A‐site cation from A^3+^ to A^+^ for an ideal cubic perovskite structure, i.e., A^III^B^III^O_3_. The dissimilar behavior of ϕ_A_ is thought to arise from the different perceptions of the Ca_3_WO_6_ structure. On the one hand, it is believed to be a monoclinic double perovskite with locally distorted A and B sites; however, Yoshimura et al. assumed a simple cubic ABO_3_ perovskite structure. Based on the above discussion, ϕ_A_ stabilization is caused not only by the introduction of oxygen and cation vacancies but also by the charge distribution of the decreasing (increasing) average valence of A(B)‐site ions in the double perovskite host crystal.

For the B‐site potential ϕ_B_ (Figure 8c), cal‐X and cal‐1,3 exhibited a clear destabilization trend with increasing Eu^3+^ and K^+^ concentrations. The evolution of cal‐2 was almost linear, whereas the evolutions of cal‐X and cal‐3 increased rapidly at x = 0.025 and showed an overall increase at higher dopant concentrations. The evolution of cal‐X was similar to that of cal‐3; their correlation coefficient was 0.82, indicating strong correlation. Based on the above results, ϕ_B_ destabilization may occur in accordance with scenario III, which changes the atomic positions of coordinating oxygens induced by substituting two Ca^2+^ ions with Eu^3+^ and with K^+^ of varying ionic radii.

In the evolution of ϕ_W_ (Figure 8d), cal‐X and cal‐3 exhibited decreasing trends with Eu^3+^ and K^+^ concentrations. Conversely, cal‐1 did not change ϕ_W_, whereas cal‐2 slightly increased ϕ_*W*_ with increasing Eu^3+^ and K^+^ concentrations. Cal‐X and cal‐3 exhibited a robust positive correlation of 0.99, indicating that ϕ_W_ stabilization must also be caused by a change in atomic position.

Considering ϕ_*i*_ evolution, which is distinguished by three scenarios, the following question is raised: What is the dominant factor affecting the evolution of each site ϕ_*i*_? It can be answered by obtaining ϕ_A_ from the charge distribution, which depends on the Eu^3+^ sites and cation/anion vacancies, and obtaining ϕ_B_ and ϕ_W_ from the atomic positions because they are more intense than those shown in Figure S3c (Supporting Information).

**Figure 9:**
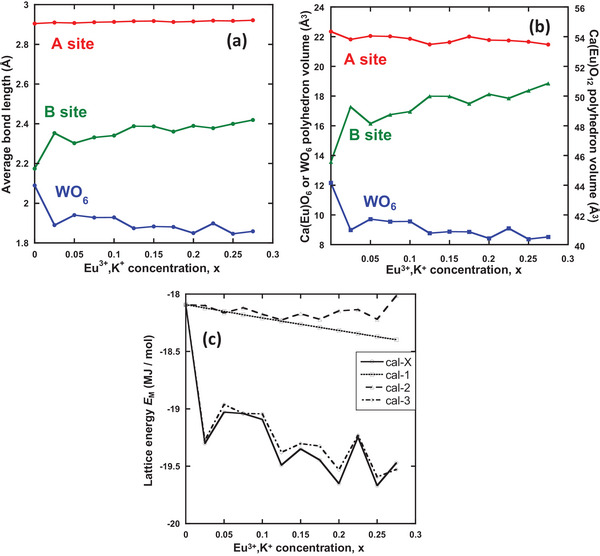
a) Average cation–oxygen bond length and b) volume of each site polyhedron (Ca(Eu)O_6_ for B site; WO_6_ and Ca(Eu,K)O_12_ for A site) as a function of Eu^3+^ and K^+^ concentrations. c) Lattice energy calculated under cal‐X or cal‐1–3 as a function of Eu^3+^ and K^+^ concentrations.

**Figure** **9**a,b shows the average bond lengths and volumes of the A‐site Ca(Eu,K)O_12_, B‐site Ca(Eu)O_6_, and WO_6_ polyhedra obtained via Rietveld refinement, which enables a better understanding of how the Ca_3_WO_6_ lattice is geometrically changed, particularly in relation to ϕ_B_ and ϕ_W_. Focusing on the average bond length, the B‐site Ca lengthened and the W‐site Ca shortened with increasing Eu^3+^ and K^+^ concentrations. In addition, the expansion of the Ca(Eu)O_6_ polyhedra and shrinkage of the WO_6_ polyhedra are shown in Figure 9b, based on the results shown in Figure 9a. The change in the polyhedral volume of site B was affected by the B‐site Eu^3+^ concentration because the concentration of Eu^3+^ ions at the B‐site exhibited a higher correlation coefficient (0.92) with the B‐site bond length. Therefore, ϕ_B_ destabilized and ϕ_W_ stabilized with increasing Eu^3+^ and K^+^ concentrations.

Interestingly, although the average bond length of site A was almost unaffected by doping and only slightly increased with Eu^3+^ and K^+^ doping, the A‐site polyhedron volume decreased with doping. Based on the results, the volume change appears to be caused by a change in corner oxygen position followed by a change in W‐ and B‐site polyhedra volumes, keeping with Ca(A)−O bond length.

The lattice energies *E*_M_ calculated using cal‐X and cal‐1–3 are shown in Figure 9c. The *E*_M_ was estimated using cal‐X and cal‐1,3 stabilized with Eu^3+^ and K^+^. The contribution to *E*_M_ was significant, decreasing in the order ϕ_W_ > ϕ_A_ > ϕ_B_ because the formal charge of W^6+^ was triple that of the B‐site Ca^2+^, whereas the A‐site Ca^2+^ ions occupied double the equivalent positions of B‐site Ca^2+^. Thus, even though ϕ_B_ was destabilized by the expansion of the B‐site polyhedron, *E*_M_ was stabilized because the ϕ_W_ and ϕ_A_ potentials were significantly stabilized by the shrinkage of the WO_6_ polyhedron and change in charge distribution by doping and oxygen defects. In conclusion, because *E*_M_ stabilization is caused by ϕ_W_ and ϕ_A_ stability induced by Eu^3+^ substitution at site B, B‐site substitution is dominant in the Eu^3+^ distribution. Furthermore, increasing the concentration of K^+^ at the A sites induces the destabilization of ϕ_B_ by the expansion of the B‐site polyhedron, which can be compensated for by decreasing the ϕ_A_ site potential.

In the following section, the geometrical evolution of A and B site structures for Eu^3+^ ions will be discussed in relation with the Eu^3+^ asymmetry ratio obtained from the luminescence data. Thus, it is worth mentioning the question of which structural model is most convincing for the Ca_3_WO_6_:Eu^3+^,K^+^ phosphors. It should be noted that the atomic ratios, including Eu^3+^ and K^+^ ions, were experimentally determined though EDS measurements. The distribution of Eu^3+^ ions between the A and B sites was estimated using the high‐resolution PLE technique of FLN, which demonstrated high reliability even for the low concentration x = 0.025, as shown in Figure 5 as well as Figure S2 (Supporting Information). The elemental information were used as fixed parameters in the subsequent Rietveld refinement, resulting in the “FLN” distribution model with satisfactory reliability factors, as presented in Figure 7b,c and Table S3 (Supporting Information). A comparison was made with two hypothetical models: one assuming 100% occupancy at the A site and another assuming 100% occupancy at the B site, based on the atomic ratio obtained through the EDS measurements. These models were then individually to Rietveld analyses, referred to as “A site” and “B site” substitution models, respectively. The increased reliability factors (*R*_wp_ and *R*_F_) were observed in both cases, indicating that the refinement using the Eu^3+^ distribution successfully reproduced the observed synchrotron XRD patterns of the samples. This practical model provides an explanation for the structure of the respective sample and will be used in the next section.

### 3.2 Geometrical Distortion Parameters and Asymmetry Ratio

**Figure 10:**
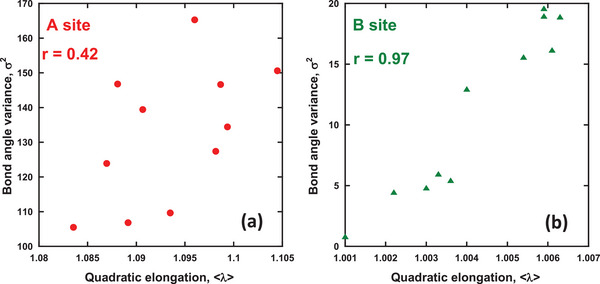
2D plots of quadratic elongation and bond‐angle variance in a) A‐site and b) B‐site polyhedra with correlation coefficient, r.

**Figure 11:**
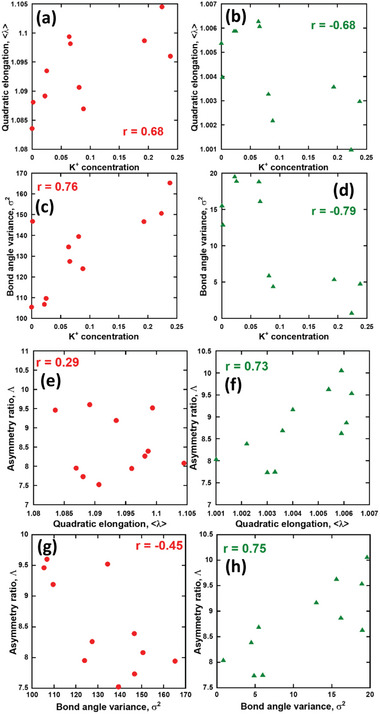
2D plots of quadratic elongation and bond‐angle variance for the a,c) A‐site and b,d) B‐site polyhedra as a function of K^+^ concentration, as experimentally estimated using EDS. e–h) Correlation of the asymmetry ratio of the e,g) A‐site and f,h) B‐site PL obtained via the FLN technique with quadratic elongation and bond‐angle variance. The correlation coefficient, r, is shown in the figure.

In general, the ^5^D_0_−^7^F_2_ ED transition of Eu^3+^ ions can be allowed for Eu^3+^ ions at lattice sites without inversion symmetry. To the best of our knowledge, parameters that appropriately describe the ED transition intensity have not yet been proposed for Eu^3+^‐doped phosphors. A geometrical parameter reflecting the asymmetry ratio Λ would help screen a variety of potential phosphors for the design of high‐color‐purity red phosphors. In this study, the representative geometrical distortion parameters of coordination polyhedra, namely quadratic elongation <λ> and bond angle variance (BAV) σ^2^, were proselytized by Robinson et al.,^[^ ^83^ ^]^ who investigated the correlation using the asymmetry ratio defined by Equation 1. The parameters <λ> and σ^2^ were calculated using Equations 11 and 12:

(11) $$\langle \lambda \rangle =\frac{1}{n}\;\sum_{i\;=\;1}^{n}{\left(\frac{l_{i}}{l_{0}}\right)}^{2}$$

(12) $$\sigma^{2}=\frac{1}{m-1}\;\sum_{i\;=\;1}^{m}{\left(\theta_{i}-\theta_{0}\right)}^{2}$$

where *n* denotes the number of all coordinate oxygen bonds with the center cation in the polyhedron; *l_i_* denotes the length from the center cation to the *i*‐th oxygen; *l*_0_ denotes the center‐to‐vertex distance for an octahedron with *O*_h_ symmetry, whose volume is equal to that of the strained or distorted octahedron with bond length *l_i_*; *m* denotes the number of angles consisting of nearest‐neighbor oxygen–cation bonds in the polyhedron; θ_*i*_ denotes the *i*‐th O–M–O angle, and θ_0_ denotes the O–M–O angle of the corresponding regular polyhedra. **Figure** **10**a,b shows the distortion parameter plots of the quadratic elongation and BAV at the A‐site cuboctahedron and B‐site octahedron, respectively, in Ca_3‐2x_Eu_x_K_x_WO_6_, with the correlation coefficient *r*. For both the A‐site cuboctahedron and B‐site octahedron, <λ> and σ^2^ were linearly plotted, where *r*_Asite_ = 0.42 and *r*_Bsite_ = 0.97, respectively. Robinson et al.^[^ ^83^ ^]^ demonstrated a strong linear correlation between <λ> and σ^2^ on the tetrahedra and octahedra of eight and five types of compounds, respectively. Another study on a variety of compounds revealed a strong relationship between the two parameters.^[^ ^84^ ^]^ Specifically, a strong linear correlation between <λ> and σ^2^ was confirmed for the B‐site octahedra of the double‐perovskite‐type compound Ca_3‐2x_Eu_x_K_x_WO_6_. To determine whether <λ> or σ^2^ was affected by the structural components, 2D plots of <λ> and σ^2^ in the A‐ and B‐site polyhedra and K^+^ ion concentrations were obtained via EDS measurements, as shown in **Figure** **11**a–d with the corresponding correlation coefficients. The 2D plots of the asymmetry ratio Λ as a function of <λ> or σ^2^ for sites A and B are shown in Figure 11e–h. The correlation coefficients between <λ> or σ^2^ and the K^+^ ion concentrations were found to be strongly positive or negative and linear at the A and B sites, respectively, indicating that K^+^ doping increased the distortion of the A‐site Ca(Eu)O_12_ cuboctahedra and decreased the distortion of the B‐site Ca(Eu)O_6_ octahedra. A similar trend was observed in previous studies on Eu^3+^‐doped double‐perovskite‐type phosphors, where the evolution of the asymmetry ratio of Eu^3+ 5^D_0_−^7^F_1,2_ transitioned with increasing concentrations of K^+^ ions, which were co‐doped in the host compound.^[^ ^7^, ^35^, ^85^ ^]^

Figure 11e–h reveal that <λ> (or σ^2^) are well correlated with the asymmetries of the B‐site octahedra, although there is no correlation between <λ> (or σ^2^) and Λ for the A‐site cuboctahedra. Notably, <λ> and σ^2^ are structurally averaged parameters representing the features of the MO_6_ and MO_12_ polyhedra (M = Ca and Eu (or K)), whereas Λ is a spectroscopically obtained parameter that reflects the local environment around the Eu^3+^ ions. Thus, the correlation coefficients for the A‐site cuboctahedra and B‐site octahedra can be interpreted as the degree to which MO_n_ polyhedral distortion coincides with that contributing to the luminescence of Eu^3+^. The linear correlations in Figure 11f,h indicate that <λ> and σ^2^ of the Ca(Eu)O_6_ octahedra appropriately describe the distortion of the B‐site EuO_6_. Conversely, no correlation between <λ> (or σ^2^) and Λ of the A‐site cuboctahedra was observed. The difference in the tendencies of the A‐ and B‐site polyhedra resulted from their rigidity, which arises from the strength of the bond between oxygen and the center cation of each polyhedral site. If the bond is covalent, the polyhedral shape is easily maintained. Conversely, if the bond is weak, the polyhedral shape is likely to change via Eu^3+^ substitution at the Ca sites. In other words, <λ> and σ^2^ of Ca(Eu)O_6_, estimated via Rietveld refinement, were similar in evolution to the Λ values spectroscopically determined for the EuO_6_ octahedra; however, the polyhedron distortion of Ca(Eu)O_12_ appeared to differ from that of the EuO_12_ polyhedron. Although further investigations using expansive compounds are required, it is noteworthy that the geometrical distortion parameters <λ> and σ^2^ could be helpful in reflecting the distortion around the Eu^3+^ ions when exploring new Eu^3+^‐doped red phosphors.

Lastly, it is worth mentioning that the effects of K^+^ co‐doping on the asymmetry ratio, quadratic elongation, and bond angle variance were discussed in the final part of this study. The evolution of luminescence properties with varying concentrations of Eu^3+^ and K^+^ was carefully tracked, and it was found that the luminescence evolution is influenced by both Eu^3+^ and K^+^ doping concentrations, encompassing all substitution effects. Therefore, an attempt was made to statistically separate and analyze the doping effects of each element. The correlation coefficient between the fraction of each element in the samples and the quadratic elongation or bond angle variance was examined. The element fractions, including Eu^3+^ and K^+^ ions, were experimentally evaluated through EDS measurements, as shown in Table S1 (Supporting Information). The statistical analysis revealed a strong correlation coefficient between the B site asymmetry ratio (Λ_*B*_) and the quadratic elongation or bond angle variance at the B site (Figure 11f,h). Additionally, there was a strong negative correlation coefficient between the quadratic elongation or bond angle variance at the B site and the concentration of K^+^ (Figure 11b,d). However, the A site asymmetry ratio (Λ_*A*_) exhibited weaker correlations with the quadratic elongation or bond angle variance at the A site (Figure 11e,g). These analytical results indicate that the asymmetry ratio at the B site is primarily affected by the K^+^ concentration as a single variable, whereas the asymmetry ratio at the A site is comparatively less influenced.

## 4 Conclusion

The optical properties and structural parameters of Ca_3‐2x_Eu_x_K_x_WO_6_ (x = 0.025–0.275) were systematically investigated. The basic luminescence properties can be summarized as follows. All the samples exhibited a pure red emission with CIE coordinates of (0.64–0.65, 0.30–0.35), which approaches the ideal red color with the help of effective Eu^3+^–Eu^3+^ interactions with an increase in x, reducing ^5^D_1,2_ emissions. The highest asymmetry ratio was 7.2 at x = 0.1 when excited at 320 nm, whereas the highest IQE was 12.3% at x = 0.1 when excited at 395 nm. Site‐selective PL spectroscopy was used to estimate the microscopic asymmetry ratios. The A‐ and B site‐selective asymmetry ratios were found to be significantly high, with values of 9.8 and 10.7 at x = 0.075 and 0.125, respectively. Peak splitting was also observed in the spectra based on the local symmetries of *C*_1_ and *C*_i_ around the Eu^3+^ ions at the A and B sites, respectively, with a break in the inversion symmetry. The high‐resolution PLE spectra revealed that Eu^3+^ had a twin distribution at sites A and B. The Eu^3+^ occupation at the B site was quantitatively estimated to be 50–75 atm%, and that at the A site increased with increasing Eu^3+^ and K^+^ concentrations. The validity of the distribution was confirmed using Rietveld refinement, and lattice energies (*E*_M_) were compared for a variety of Eu^3+^ distributions, following the results of the FLN spectra and 100% A‐ or B‐site substitution models. The Eu^3+^ site distribution was explained by the site potentials (ϕ_*i*_). The B‐site substitution of Eu^3+^ ions was dominant for lower Eu^3+^ and K^+^ concentrations despite the dopant‐induced ϕ_B_ destabilization by the expansion of the B‐site polyhedron because the simultaneous substitution of K^+^ at the A site effectively stabilized the ϕ_A_ potential. Further doping caused an increase in the A‐site Eu^3+^ occupancy and reduced the *E*_M_ energy owing not only to the stabilization effect but also the decrease in ϕ_W_, resulting in well‐structured WO_6_ frameworks. Based on these results, it was concluded that two factors of the Eu^3+^ lattice site endowed Ca_3‐2x_Eu_x_K_x_WO_6_ with an extremely high asymmetry ratio and red PL color purity: 1) the lower symmetry (higher asymmetry ratio) and higher Eu^3+^ distribution at the B site compared with those of the A site, and 2) Eu^3+^–Eu^3+^ interactions, which reduced ^5^D_1,2_ emissions. The methodology demonstrated here can be applied to Eu^3+^ crystalline phosphors with multiple Eu^3+^ sites to elucidate the structural origins of their luminescence properties and is promising for accelerating the development of new Eu^3+^ phosphors.

## 5 Experimental Section

### Sample Preparation

Powdered samples of Ca_3‐2x_Eu_x_K_x_WO_6_ (x = 0.025–0.275) were prepared using a conventional solid‐state reaction method. The raw materials were CaCO_3_ (Rare Metallic Co. Ltd., 99.99%), WO_3_ (Nacalai Tesque, 99.5%), Eu_2_O_3_ (Shin‐Etsu Chem. Co., 99.99%), and K_2_CO_3_ (Kishida Chem. Co., 99.5%). All the raw materials were mixed in stoichiometric amounts for 20 min in an agate mortar. The mixed powders were then placed in an aluminum crucible and calcined at 1200 °C in air for 12 h.

### Characterization

XRD patterns were obtained to confirm the crystal phases of the samples using an X‐ray diffractometer (PANalytical X'pert Pro) with Cu Kα radiation (λ = 1.5418 Å) at a tube voltage of 45 kV and tube current of 40 mA. Synchrotron XRD measurements of the samples were performed to obtain the crystal structure parameters at the BL5S2 beamline of the Aichi Synchrotron Radiation Center (Aichi Science & Technology Foundation, Aichi, Japan). The X‐ray wavelength was 0.8002 Å. Rietveld refinement was performed using the RIETAN‐FP.^[^ ^86^ ^]^ The Madelung electrostatic site potential and lattice energy were calculated from the crystal structure using MADEL, a Madelung potential calculation software built into VESTA.^[^ ^82^ ^]^ The Eu^3+^ concentrations of the samples were estimated using energy‐dispersive X‐ray spectroscopy (EDS) using a JEOL JSM‐6010LA scanning electron microscope (SEM) with an acceleration voltage of 10 kV. The photoluminescence (PL) and photoluminescence emission (PLE) spectra were measured using a Hitachi F‐7000 fluorescence spectrophotometer. In addition, the quantum efficiency was assessed using a spectrophotometer with a BaSO_4_‐coated integration sphere. The FLN spectra were obtained using a tunable dye laser (rhodamine 6G:566–640 nm) pumped by an Ar^+^ laser (Coherent, Innova 70). The dye laser with a linewidth of ≈1 cm^−1^ could site‐selectively excite Eu^3+^ ions within the inhomogeneous absorption width of the ^7^F_0_–^5^D_0_ transition.^[^ ^31^ ^]^ During the acquisition of the PLE spectra, a larger slit width corresponding to ≈15 nm was used to collect the widest possible ^5^D_0_‐^7^F_2_ luminescence component, which ranged from 600 to 630 nm.

## Conflict of Interest

The authors declare no conflict of interest.

## Author Contributions

T.O. contributed in conceptualization, data curation, formal analysis, investigation, wrote – original draft, and visualization. R.O. contributed in writing‐review and editing, investigation, methodology, and validation. T.H. contributed in conceptualization, methodology, software, resources, investigation, writing‐review and editing, visualization, supervision, funding acquisition, validation, and project administration.

## Supporting information

Supporting Information

## Data Availability

The data that support the findings of this study are available in the supplementary material of this article.
