# Supplementary material for: Eu3+ Site Distribution and Local Distortion of Photoluminescent Ca3WO6:(Eu3+, K+) Double Perovskites as High‐Color‐Purity Red Phosphors
Source: Adv Sci (Weinh). 2023 Sep 26;10(31):2302559. doi: 10.1002/advs.202302559 (PMC10625125; doi:10.1002/advs.202302559)
Supplement: Supplementary file 1 — Supporting Information [file ADVS-10-2302559-s001.pdf]

ADVANCED SCIENCE
Open Access

Supporting Information

for *Adv. Sci.*, DOI 10.1002/advs.202302559

Eu^{3+} Site Distribution and Local Distortion of Photoluminescent Ca_3WO_6:(Eu^{3+}, K^+) Double Perovskites as High-Color-Purity Red Phosphors

*Takahito Otsuka, Ryohei Oka and Tomokatsu Hayakawa**

Supporting Information

Eu^{3+} site distribution and local distortion of photoluminescent Ca_3WO_6:(Eu^{3+}, K^+) double perovskites as high-color-purity red phosphors

Takahito Otsuka,[1,] Ryohei Oka,[1] and Tomokatsu Hayakawa[1,2]*

[1] Field of Advanced Ceramics, Life science and Applied Chemistry Department, Nagoya Institute of Technology (NITech), Japan
[2] Frontier Research Institute for Material Science (FRIMS), Nagoya Institute of Technology (NITech), Japan

1. Absorption ratios obtained in quantum efficiency measurements

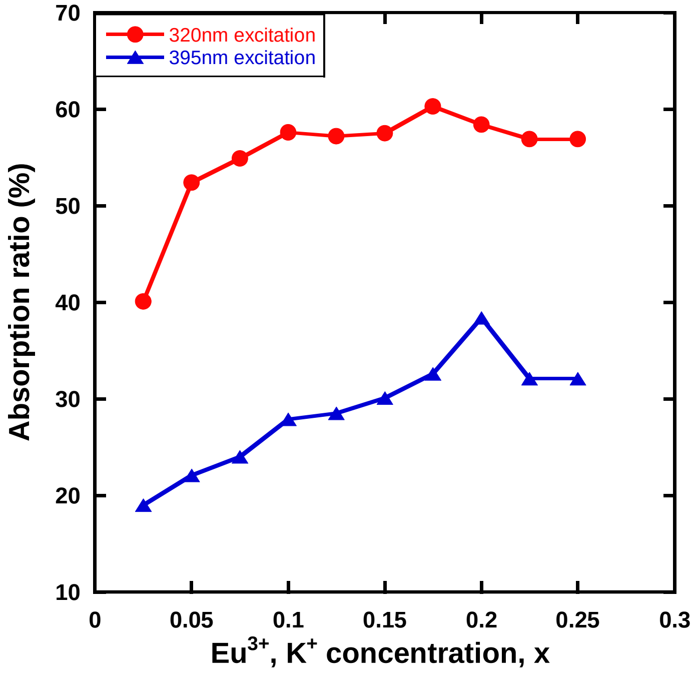

Figure S1. Absorption ratios under 320 nm and 395 nm excitations for Eu^{3+} red luminescence, defined as the number of absorption photons divided by the number of incident photons.

In the quantum efficiency measurements, the incident, absorbed, and emitted photons were measured using an integration sphere.[S1] For the detection of incident photons, a non-absorbing and well-scattering white reference (Spectralon®) was used and placed in a sample position in a side of the sphere. The incident light was diffusely reflected onto the reference and detected after multiple reflections inside the sphere (N_{ex}^{ref}). The reference was then replaced with a powdered sample stored in a sample holder, and the sample was irradiated with incident light. Some of the incident photons were absorbed by the sample, while others were reflected and detected after multiple reflections in the sphere (N_{ex}^{smpl}), similar to the reference. The difference in the detected signal of the sample and that of the reference is proportional to the number of absorbed photons (N_{abs}). The energy absorbed by the sample can be converted to photoluminescence after

nonradiative relaxation to an emissive level, which is also multireflected in the sphere in the same manner as in the case of incident light, and the number of emitted photons is detected (N_{em}).

$$N_{abs} = N_{ex}^{ref} - N_{ex}^{smpl} \tag{S1}$$

$$IQE = \frac{N_{em}}{N_{abs}} = \frac{N_{em}}{N_{ex}^{ref} - N_{ex}^{smpl}} \tag{S2}$$

$$EQE = \frac{N_{em}}{N_{ex}^{ref}} \tag{S3}$$

The internal and external quantum efficiencies were defined as the ratios of the numbers of the emitted/absorbed photons and the emitted/irradiated photons, respectively. The absorption ratio was defined as the ratio of the number of absorbed photons to the number of irradiated photons.

$$Absorption\ ratio = \frac{N_{abs}}{N_{ex}^{ref}} = \frac{EQE}{IQE} \tag{S4}$$

Figure S1 shows the absorption ratio of Ca_3WO_6:x(Eu^{3+},K^+) phosphors as a function of the Eu^{3+},K^+ concentration, x, under 320 nm (CTB) and 395 nm (*f–f* direct) excitations. It was found that the absorption ratio increased with x for the 395 nm excitation, while it rapidly increased and maintained a constant value for the 320 nm excitation. The 320 nm (CTB) excitation shows higher absorption ratios than the 395 nm (*f–f* direct) excitation, which could be due to the influence of various non-radiative relaxation processes down to the emissive level ($^5D_0$), as well as host absorption, as discussed in the following section.

2. EDS elementary analysis for $Ca_{2-2x}Eu_xK_xWO_6$ phosphors

Table S1. EDS results for $Ca_{2-2x}Eu_xK_xWO_6$ samples.

x		Ca		W		Eu		K		O	
0.025	Theoretical	29.5		10		0.25		0.25		60.00	
	Experimental	33.155	±0.071	11.48	±0.14	0.41	±0.10	0.020	±0.021	54.945	±0.025
0.050	Theoretical	29		10		0.5		0.5		60.00	
	Experimental	32.790	±0.058	11.51	±0.12	0.687	±0.087	0.000	±0.030	54.980	±0.023
0.075	Theoretical	28.5		10		0.75		0.75		60.00	
	Experimental	31.750	±0.046	11.92	±0.10	0.993	±0.075	0.263	±0.017	55.077	±0.017
0.100	Theoretical	28		10		1		1		60.00	
	Experimental	30.537	±0.052	11.62	±0.11	1.310	±0.085	0.290	±0.017	56.243	±0.017
0.125	Theoretical	27.5		10		1.25		1.25		60.00	
	Experimental	30.983	±0.052	12.02	±0.10	1.863	±0.087	0.77	±0.017	54.363	±0.017
0.150	Theoretical	27		10		1.5		1.5		60.00	
	Experimental	30.280	±0.046	12.22	±0.10	1.907	±0.077	0.803	±0.017	54.790	±0.017
0.175	Theoretical	26.5		10		1.75		1.75		60.00	
	Experimental	29.743	±0.052	11.64	±0.11	1.877	±0.094	1.027	±0.021	55.660	±0.019
0.200	Theoretical	26		10		2		2		60.00	
	Experimental	29.957	±0.054	11.54	±0.12	2.03	±0.10	0.930	±0.021	56.210	±0.021

0.225	Theoretical	25.5		10		2.25		2.25		60.00	
	Experimental	24.547	±0.056	10.46	±0.12	1.96	±0.11	2.020	±0.023	61.020	±0.023
0.250	Theoretical	25		10		2.5		2.5		60.00	
	Experimental	26.440	±0.052	11.47	±0.11	2.57	±0.094	2.733	±0.023	56.787	±0.019
0.275	Theoretical	24.5		10		2.75		2.75		60.00	
	Experimental	25.280	±0.046	11.96	±0.11	2.84	±0.092	2.660	±0.023	55.943	±0.017
0.300	Theoretical	24		10		3		3		60.00	
	Experimental	22.483	±0.048	11.42	±0.11	2.78	±0.10	5.777	±0.029	56.497	±0.019

The EDS results are listed in **Table S1**. The experimentally observed [Ca]/[W] ratios were 2.888 (x =0.025), 2.849 (x=0.050), 2.664 (x=0.075), 2.628 (x=0.100), 2.578 (x=0.125), 2.478 (x=0.150), 2.555 (x=0.175), 2.596 (x=0.200), x=2.347 (x=0.225), 2.305 (x=0.250), 2.114 (x=0.275), and 1.969 (x=0.300), demonstrating a monotonical decrease of Ca content owing to Eu^{3+} and K^+ substitution, except for x=0.175 and 0.200.

3. Color purity as a function of the asymmetry ratio

Table S2. CIE coordinates (CIE x, y), color purity (*C.P.*), and asymmetry ratio (Λ) of various Eu^{3+}-doped phosphors..

Composition	Abbr.	CIE x	CIE y	*C.P.*	Λ	$Log_{10}(\Lambda)$	Ref.*
$Y_{2.97}Al_5O_{12}:0.03Eu^{3+}$	YAG	(0.53,	0.45)	68.6	0.27	-0.569	[57]
$Y_{0.8}BO_3:0.2Eu^{3+}$	YBO	(0.65,	0.35)	91.1	2.2	0.342	[58]
$Y_{0.95}AlO_3:0.05Eu^{3+}$	YAO	(0.645,	0.353)	89.8	1.54	0.189	[59]
$Y_{0.95}VO_4:0.05Eu^{3+}$	YVO	(0.63,	0.37)	86.5	4.29	0.632	[60]
$Y_{1.998}O_3:0.002Eu^{3+}$	YO	(0.655,	0.322)	92.0	5.39	0.731	[61]
$Y_{1.88}O_2S:0.12Eu^{3+}$	YOS	(0.665,	0.334)	94.8	6.26	0.797	[62]
$Ba_{0.94}TiO_3:0.06Eu^{3+}$	BTO	(0.62,	0.36)	83.5	0.93	-0.032	[63]
$BaZr_{0.97}O_3:0.03Eu^{3+}$	BZO	(0.574,	0.425)	76.1	0.40	-0.398	[64]
$Sr_{0.97}ZrO_3:0.03Eu^{3+}$	SZO	(0.622,	0.378)	84.8	2.46	0.392	[64]
$Ca_2Y_{0.6}NbO_6:0.4Eu^{3+}$	CYNO	(0.653,	0.346)	91.9	4.54	0.657	[65]
$Sr_{2.4}WO_6:0.3(K^+,Eu^{3+})$	SWO	(0.66,	0.34)	93.5	6.20	0.792	[15]
$Li_3Ba_2Gd_{0.6}Eu_{2.4}(MoO_4)_8$	LBGMO	(0.67,	0.33)	96.0	8.10	0.908	[66]
$Ba_3(Bi_{0.5}Eu_{0.5})_2(BO_3)_4$	BBBO	(0.65,	0.35)	91.1	2.62	0.419	[67]
$Y_{1.95}SiO_5:0.05Eu^{3+}$	YSO	(0.65,	0.31)	90.7	2.44	0.387	[68]
$CaGd_{0.93}AlO_4:0.07Eu^{3+}$	CGAO	(0.641,	0.358)	88.9	2.46	0.391	[69]
$Ca_{9.9}Li(PO_4)_7:0.1Eu^{3+}$	CLPO	(0.638,	0.361)	88.3	2.78	0.443	[70]
(Correlation Coefficient)					(0.750)	(0.917)	

*Reference number corresponds to that given in the main text.

4. Deconvoluted high-resolution PLE spectra

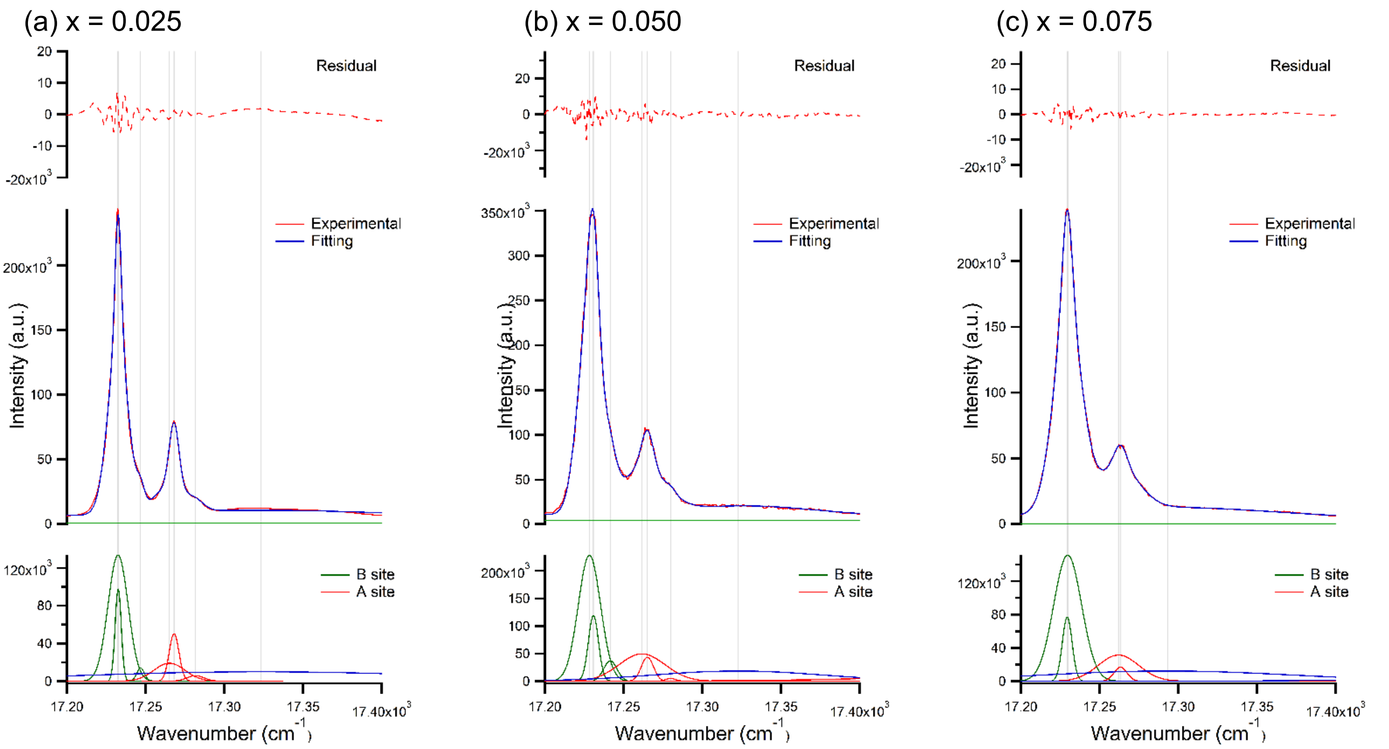

Figure S2. a–c) Deconvoluted high-resolution PLE spectra for $Ca_{2-2x}WO_6$:x(Eu^{3+}, K^+) monitored at 615 nm for low Eu^{3+} concentrations of x = 0.025–0.075.

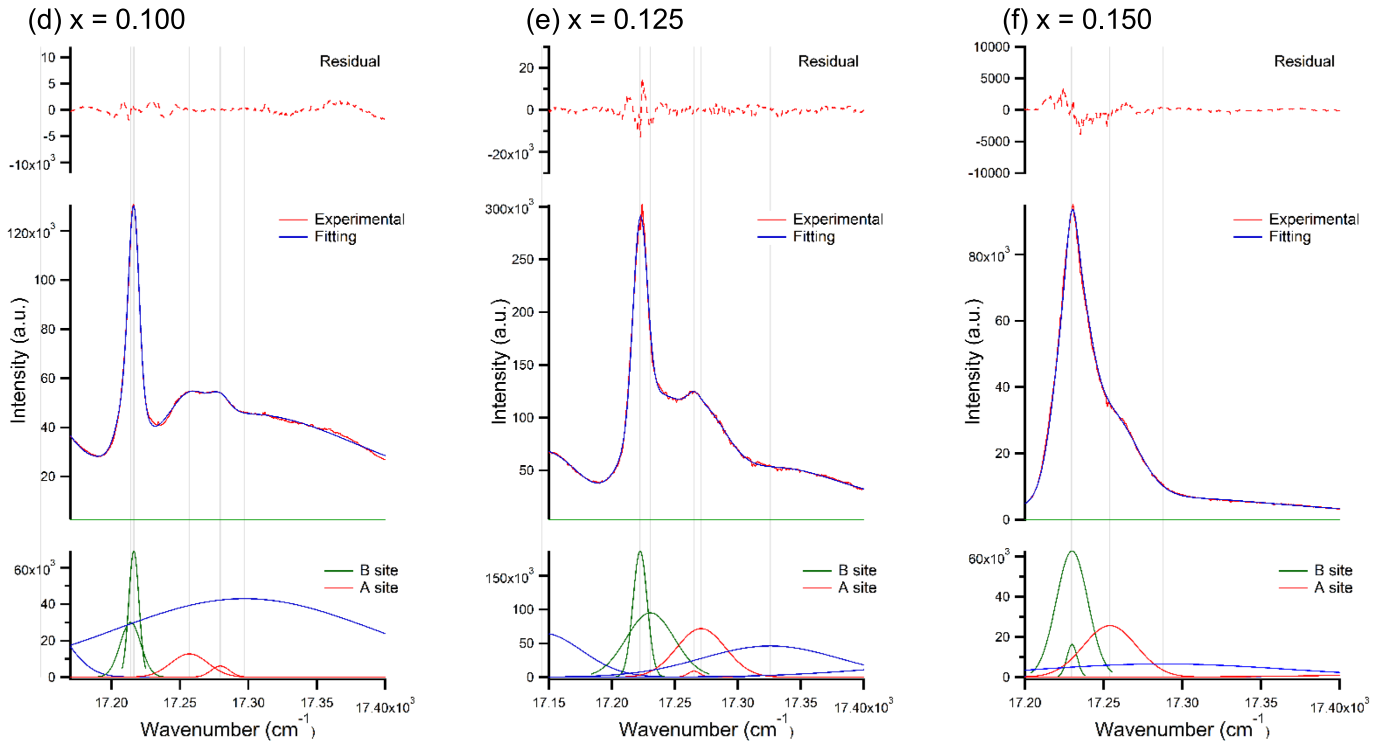

Figure S2. d–f) Deconvoluted high-resolution PLE spectra for $Ca_{2-2x}WO_6$:x(Eu^{3+}, K^+) monitored at 615 nm for low Eu^{3+} concentrations of x = 0.100–0.150.

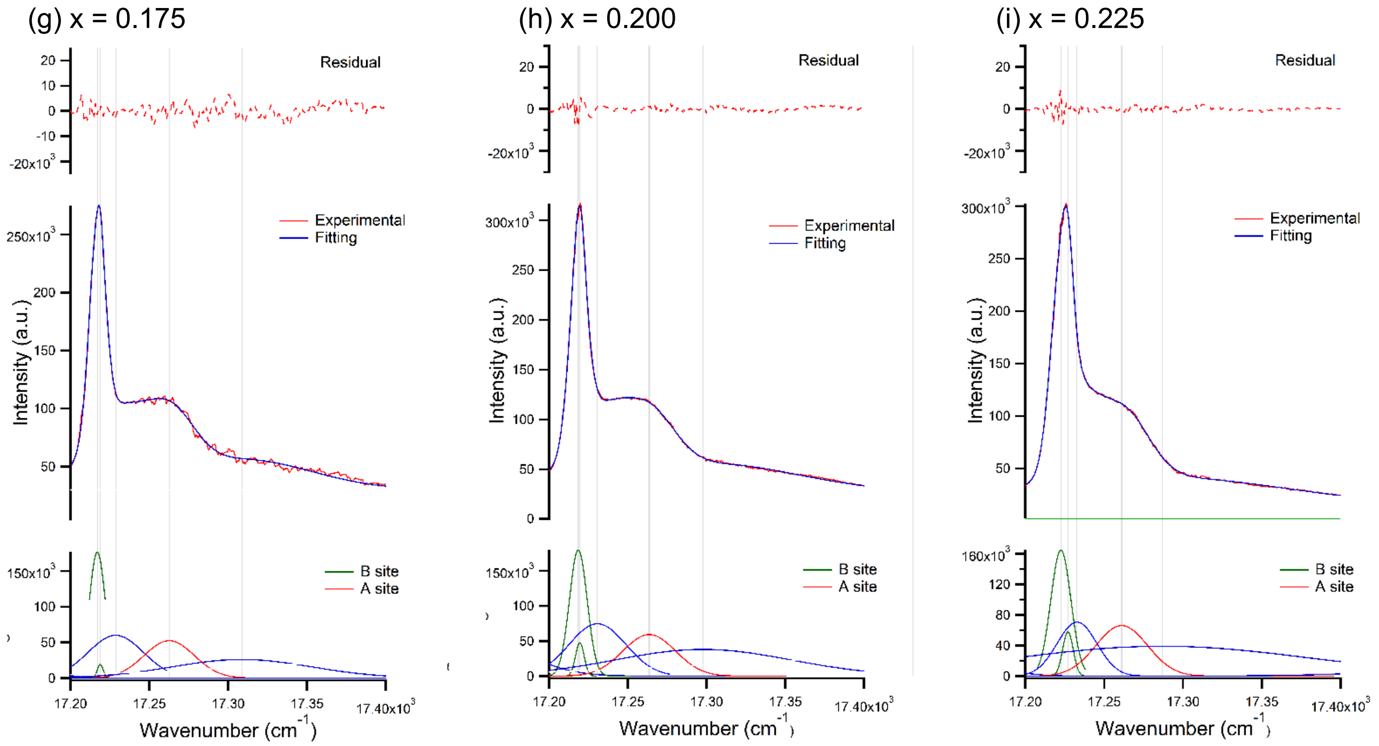

Figure S2. g–i) Deconvoluted high-resolution PLE spectra of $Ca_{2-2x}WO_6$:x(Eu^{3+}, K^+) monitored at 615 nm for high Eu^{3+} concentrations of x = 0.175–0.225.

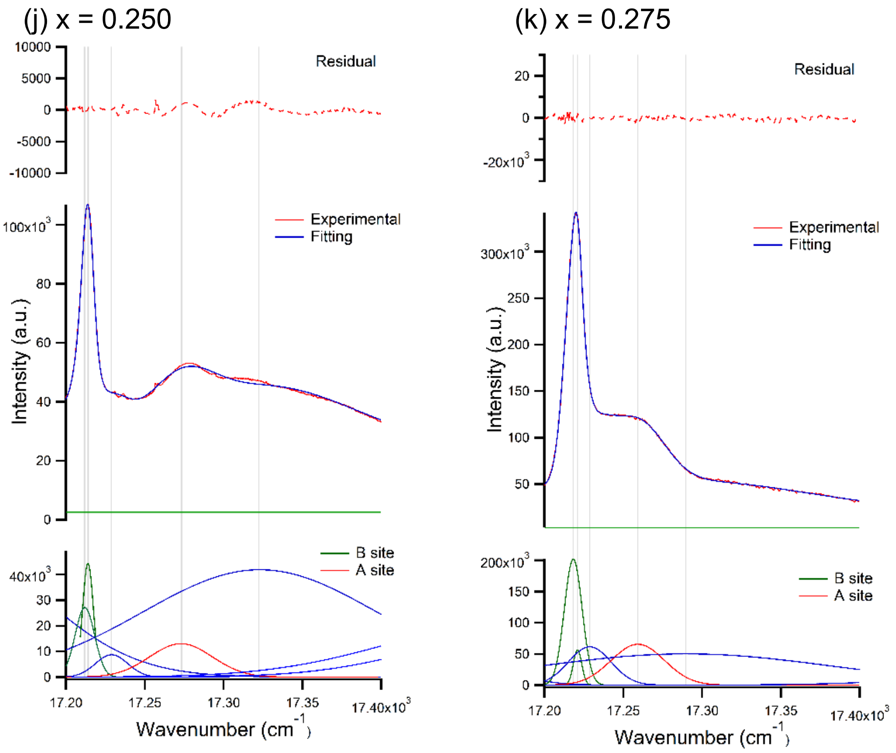

Figure S2. j–k) Deconvoluted high-resolution PLE spectra of $Ca_{2-2x}WO_6$:x(Eu^{3+}, K^+) monitored at 615 nm for high Eu^{3+} concentrations of x = 0.250–0.275.

All the deconvoluted PLE spectra obtained using the FLN technique are given in **Figure S2**a (x=0.025), b (x=0.050), c (x=0.075), d (x=0.100), e (x=0.125), f (x=0.150), g (x=0.175), h (x=0.200), i (x=0.225), j (x=0.250), and k (x=0.275). The fitting results are satisfactory. For low Eu^{3+} doping concentrations, two sharp excitation peaks were observed and assigned to sites A and B at lower and higher energy positions, respectively (see main text). To count the contributions of the respective PL excitations, the deconvolutions were performed in a wavenumber vs. PL intensity graph, which led to the results of **Figure 7a** (main text). The experimental PLE spectra given in the main text are shown in a wavelength vs PL intensity graph, together with its deconvolution data. Besides the two peaks, broader contributions in the PLE spectra are observed, whose possible origins are discussed in the main text.

5. Fundamentals of estimation of Eu^{3+} distribution in multiple sites

First, let us consider the $^5D_0$–^7F_J photoluminescence of Eu^{3+} ions in a single site. The Eu^{3+} PL intensity of $^5D_0$–^7F_J emission is proportional to the population of Eu^{3+} ions in the emissive level of the $^5D_0$ excited state (N_0) and branching ratio (β_{0-J}).

$$I_{0-J} \propto h\nu_{0-J}\beta_{0-J}N_0\,, \tag{S5}$$

where $h\nu_{0-J}$ is the transition energy from the $^5D_0$ level to ^7F_J level. Assume that the population of the excited state is proportional to the number of Eu^{3+} dopant ions regardless of their local environment at the first approximation. The $^5D_0$ population was partitioned via a luminescence process into the photons of the $^5D_0$–^7F_J (0-J) transitions. The most intense emissions were obtained from the $^5D_0$–$^7F_1$ (0-1) and $^5D_0$–$^7F_2$ (0-2) transitions, and thus were selected because the 0-0 and 0-3,4,5,6 transitions were sufficiently weak in comparison. The branching ratio of the 0-2 transition can be expressed as the ratio of the transition probability W_{0-2} to the sum of the total transition probabilities:

$$\beta_{0-2} = \frac{W_{0-2}}{W_{0-1} + W_{0-2}} \tag{S6}$$

The energy gap between the $^5D_0$ level and the next energy level below $^5D_0$ is very large (≈12.300 cm^{-1}), and thus the multiphonon relaxation process from the $^5D_0$ level is negligible. The asymmetry ratio given in the main text can be expressed in the following equation and approximated as the ratio of the transition probabilities W_{0-2} and W_{0-1}.

$$\begin{aligned}\Lambda = \frac{I_{0-2}}{I_{0-1}} &= \frac{h\nu_{0-2}W_{0-2}}{h\nu_{0-J}W_{0-J}} \\ &= 0.967\frac{W_{0-2}}{W_{0-J}} \\ &\sim \frac{W_{0-2}}{W_{0-J}}\end{aligned} \tag{S7}$$

From **Equation S6** and **S7**, the branching ratio for the $^5D_0$–$^7F_2$ (0-2) transition is given by

$$\beta_{0-2} = \frac{\Lambda}{1+\Lambda}. \tag{S8}$$

Even if the approximation in Equation S7 is applied, for Λ ~10, the deviation of the branching ratio is estimated to be less than 1%.

In a two-site model, such as A and B sites in a double perovskite structure, Eu^{3+} ions in the respective sites can emit luminescence with different 0-2 transition probabilities, W_{0-2}^{A} and W_{0-2}^{B}, where the 0-1 transition probability is independent of the environment owing to the nature of the magnetic dipole, $W_{0-1}^{A} = W_{0-1}^{B} = W_{0-1}$ (see the main text). The $^5D_0$-$^7F_2$ PL intensities from different Eu^{3+} sites (A and B) are given by the following equations: [S2]

$$I_{0-2}^{A} = c_{Inst} h\nu_{0-2} \beta_{0-2}^{A} N_0^{A}, \quad \text{(S9)}$$

$$I_{0-2}^{B} = c_{Inst} h\nu_{0-2} \beta_{0-2}^{B} N_0^{B}, \quad \text{(S10)}$$

where c_{Inst} is a constant related to the optical-detection system. If the respective emissions are distinguishable, the Eu^{3+} distributions for sites A and B can be estimated using the following equation:

$$P_A = \frac{N_0^A}{N_0^A + N_0^B} = \frac{\frac{I_{0-2}^A}{\beta_{0-2}^A}}{\frac{I_{0-2}^A}{\beta_{0-2}^A} + \frac{I_{0-2}^B}{\beta_{0-2}^B}}, \quad \text{(S11)}$$

$$P_B = \frac{N_0^B}{N_0^A + N_0^B} = \frac{\frac{I_{0-2}^B}{\beta_{0-2}^B}}{\frac{I_{0-2}^A}{\beta_{0-2}^A} + \frac{I_{0-2}^B}{\beta_{0-2}^B}}, \quad \text{(S12)}$$

$$\beta_{0-2}^{A} = \frac{\Lambda_A}{1 + \Lambda_A}, \quad \text{(S13)}$$

$$\beta_{0-2}^{B} = \frac{\Lambda_B}{1 + \Lambda_B}, \quad \text{(S14)}$$

These equations are identical to Equation 5–8 during the maintenance phase. The branching ratio $\beta_{0-2}^{A,B}$ plays a role in the calibration factors for the $^5D_0$-$^7F_2$ emission from the respective site.

6. Reliability factors of Rietveld analysis

In **Table S3**, reliability factors of R_{wp} and R_F of the Rietveld analysis using for the experimental atomic ratios and Eu^{3+} distribution, called a "FLN" model, were compared with two hypothetical models of 100% A site and 100% B site, which were used in individual Rietveld analyses, denoted with a subscription of "A site" and "B site", The resultant reliability factors were increased in comparison the "FLN" model. The R_e factor in the Rietveld analysis for the "FLN" distribution model is also shown in **Figure 7b** and **Table S3**, that is sufficiently low, $R_e < 10$.

Table S3. R_{wp}, R_e, and R_F factors resulted from Rietveld refinement for $Ca_{2-2x}Eu_xK_xWO_6$ samples.

x	0.025	0.05	0.075	0.1	0.125	0.15	0.175	0.2	0.225	0.25	0.275
$R_{wp\ A\ site}$	6.78	7.08	7.63	7.65	7.12	8.59	8.56	7.30	8.50	8.48	9.05
$R_{wp\ B\ site}$	7.16	8.08	8.38	8.99	8.75	7.77	8.84	8.72	9.22	9.20	10.00
$R_{wp\ FLN}$	5.82	6.98	6.78	6.67	6.43	6.11	6.08	6.56	7.79	7.62	8.37
R_e	5.78	6.48	6.53	6.37	6.52	6.41	6.12	6.55	6.27	6.38	6.32
$R_{F\ A\ site}$	8.60	13.10	12.97	14.61	9.31	9.52	8.99	9.64	11.27	11.0	11.19

$R_{F\ B\ site}$	8.42	13.65	13.63	15.42	10.79	9.21	9.47	10.0	11.51	11.15	11.74
$R_{F\ FLN}$	4.25	4.86	4.85	5.98	3.30	4.37	8.29	5.99	11.05	8.80	7.56

7. Electrostatic site potential and Madelung lattice energy calculation

The electrostatic site potentials and Madelung lattice energies calculated using the VESTA software[S3] are given in **Figure S3** under three sets of parameters: Eu^{3+} occupation, chemical composition, and atomic positions, as listed in **Table S4**. The FLN results presented here are the same as those for cal-1, as shown in the main text.

Table S4. Adopted parameters for electrostatic site potential and Madelung lattice energy calculation.

	Eu^{3+} occupation	Chemical composition	Atomic positions
A site	A site 100%	$Ca(A)_{1-2x}K(A)_xEu(A)_xCa_2WO_6$	from database*
B site	B site 100%	$Ca(A)_{1-x}K(A)_xCa(B)_{2-x}Eu(B)_xWO_6$	from database*
FLN	FLN	$Ca_{3-2x}K(A)_xEu(A,B)_xWO_6$	from database*

* JCPDS 22-0541

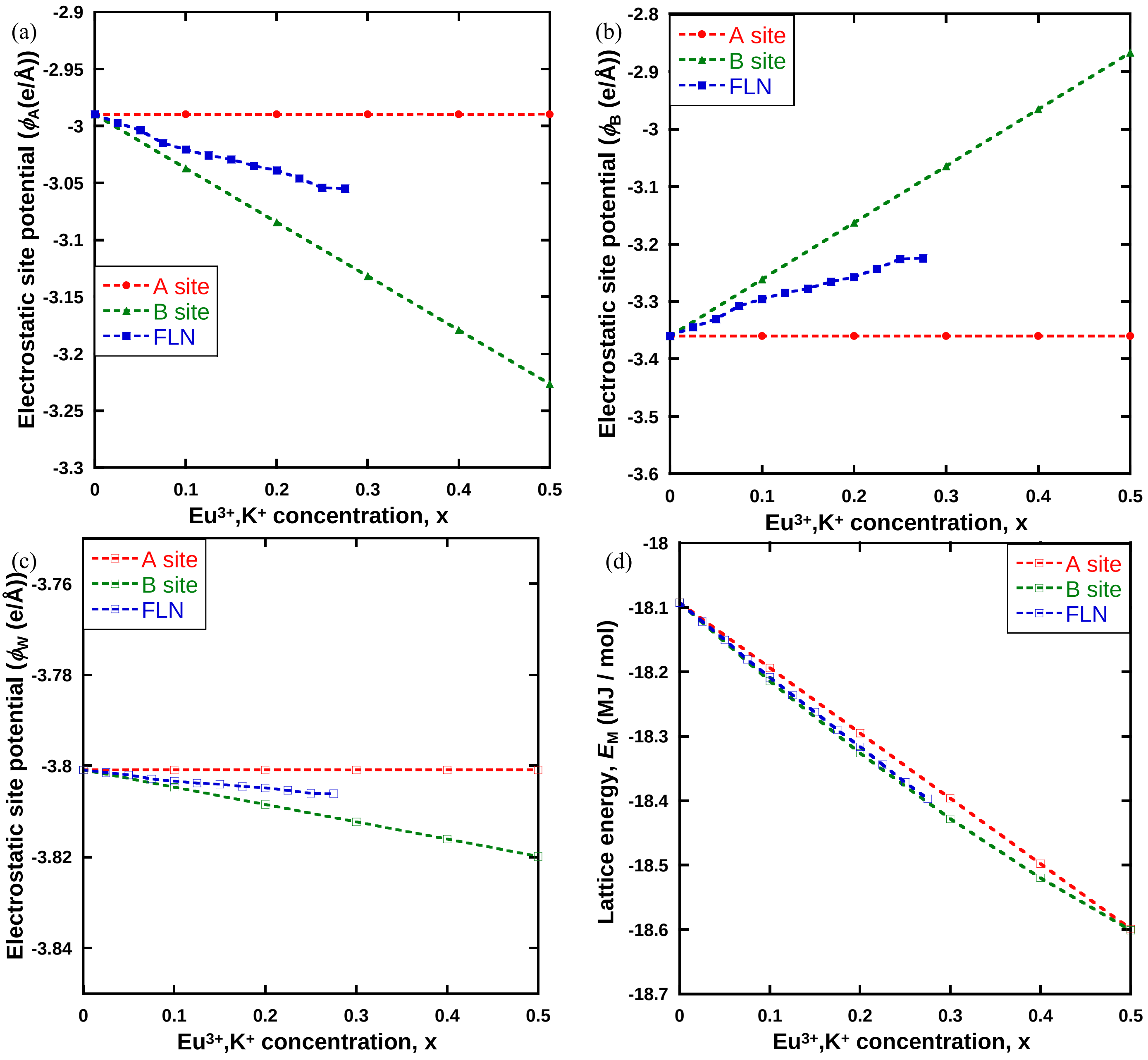

Figure S3. Electrostatic site potentials of a) Ca(Eu,K)O_{12} A-site, b) Ca(Eu)O_6 B-site, c) WO_6 site, and d) Madelung lattice energy E_M estimated under the conditions given in **Table S3**.

References

[S1] F.R.Lipsett, "The quantum efficiency of luminescence", *Progr. Dielectronics*, **7** (1967) 217-319.

[S2] T.Hayakawa, R.Ikeshita, J.-R. Duclère, A. Lecomte, "Simple method to estimate fractional numbers of Eu^{3+} ions in different phases in highly luminescent ZrO_2–SiO_2 nanocomposites", *Phys. Status Solidi B* **2022**, 2100560.

[S3] K. Momma and F. Izumi, "VESTA 3 for three-dimensional visualization of crystal, volumetric and morphology data", *J Appl Crystallogr,* **44** (2011) 1272–1276.
